# Scopoletin 8-hydroxylase: a novel enzyme involved in coumarin biosynthesis and iron-deficiency responses in Arabidopsis

**DOI:** 10.1093/jxb/ery005

**Published:** 2018-01-18

**Authors:** Joanna Siwinska, Kinga Siatkowska, Alexandre Olry, Jeremy Grosjean, Alain Hehn, Frederic Bourgaud, Andrew A Meharg, Manus Carey, Ewa Lojkowska, Anna Ihnatowicz

**Affiliations:** 1 Intercollegiate Faculty of Biotechnology of University of Gdansk and Medical University of Gdansk, Abrahama, Gdansk, Poland; 2 Université de Lorraine, INRA, UMR 1121 Laboratoire Agronomie et Environnement Nancy-Colmar, Vandœuvre-lès-Nancy, France; 3 Institute for Global Food Security, Queen’s University Belfast, David Keir Building, Malone Road, Belfast, UK

**Keywords:** Abiotic stress, Arabidopsis, enzyme activity, fraxetin, Fe- and 2OG-dependent dioxygenase, mineral nutrition, plant–environment interactions

## Abstract

Iron deficiency is a serious agricultural problem, particularly in alkaline soils. Secretion of coumarins by *Arabidopsis thaliana* roots is induced under iron deficiency. An essential enzyme for the biosynthesis of the major Arabidopsis coumarins, scopoletin and its derivatives, is Feruloyl-CoA 6′-Hydroxylase1 (F6′H1), which belongs to a large enzyme family of the 2-oxoglutarate and Fe^2+^-dependent dioxygenases. We have functionally characterized another enzyme of this family, which is a close homologue of F6′H1 and is encoded by a strongly iron-responsive gene, *At3g12900*. We purified At3g12900 protein heterologously expressed in *Escherichia coli* and demonstrated that it is involved in the conversion of scopoletin into fraxetin, via hydroxylation at the C8 position, and that it thus functions as a scopoletin 8-hydroxylase (S8H). Its function in plant cells was confirmed by the transient expression of S8H protein in *Nicotiana benthamiana* leaves, followed by metabolite profiling and biochemical and ionomic characterization of Arabidopsis *s8h* knockout lines grown under various iron regimes. Our results indicate that S8H is involved in coumarin biosynthesis, as part of mechanisms used by plants to assimilate iron.

## Teaser

A strongly iron-responsive gene of previously unknown function, *At3g12900*, encodes a scopoletin 8-hydroxylase involved in coumarin biosynthesis, and plays an important role in the iron uptake strategy of Arabidopsis.

## Introduction

Iron (Fe) is an essential micronutrient for all living organisms. In plants, chloroplasts and mitochondria have high Fe requirements (Nouet *et al.*, 2011). Fe is abundant in soils, but its availability is often limited due to soil conditions, which can be highly heterogeneous, such as the pH and redox presence of co-elements (Moosavi and Ronaghi, 2011; Marschner, 2012). Fe deficiency is a serious agricultural problem, particularly in alkaline and calcareous soils (Mengel, 1994). These types of soils, which represent approximately 30% of the world’s cropland, are characterized by a higher pH, which, in combination with the presence of oxygen, leads to precipitation of Fe in the form of insoluble ferric oxides (Fe_2_O_3_) (Morrissey and Guerinot, 2009).

Higher plants have developed two different types of strategies in response to Fe limitation. The first (Strategy I), which occurs in all plants except graminaceous species, is a reduction-based strategy, where the release of protons into the rhizosphere is enhanced by Fe-deficiency-induced proton-translocating adenosine triphosphatases (Marschner and Roemheld, 1994; Kim and Guerinot, 2007), such as AHA2 in Arabidopsis (Santi and Schmidt, 2009). As a result, at lower pH the ferric oxide precipitates are dissolved, and plasma-membrane-bound Ferric Chelate Reductase 2 (FRO2) (Robinson *et al.*, 1999) catalyses the reduction of ferric ions (Fe^3+^) into more soluble, and bioavailable to plants, ferrous ions (Fe^2+^). Fe^2+^ ions are then transported across the plasma membrane into the root epidermal cells by the divalent metal transporter Iron Regulated Transporter 1 (IRT1) (Varotto *et al.*, 2002; Vert *et al.*, 2002). A second strategy (Strategy II), used by graminaceous species, is based on the release of soluble mugineic acid family phytosiderophores (PS) from the root epidermis, which form complexes with Fe^3+^ (Takagi *et al.*, 1984; Marschner and Roemheld, 1994; Kim and Guerinot, 2007). The resulting Fe^3+^–PS complexes are then transported into the root epidermis via a high-affinity uptake system without requiring a reduction step (Curie *et al.*, 2001; Kim and Guerinot, 2007).

The precise mechanisms underlying the responses of Strategy I plants to the low Fe availability in calcareous soils are not clear. However, it is well documented that Fe deficiency enhances the release of reductants/chelators (mainly phenolics) in many dicots (Marschner and Roemheld, 1994; Jin *et al.*, 2007). Recently, it was shown that Fe deficiency induces the secretion of secondary metabolites, such as scopoletin and its derivatives, by Arabidopsis roots (Rodríguez-Celma *et al.*, 2013*a*; Fourcroy *et al.*, 2014), and that a Feruloyl-CoA 6′-Hydroxylase1 (F6′H1) is required for the biosynthesis of the Fe^3+^-chelating coumarin esculetin, which is released into the rhizosphere as part of Strategy I-type Fe acquisition (Schmid *et al.*, 2014).

Scopoletin and its corresponding glycoside, scopolin, are the predominant coumarins in Arabidopsis roots. However, many other coumarin compounds, such as skimmin, esculetin, fraxetin, and the recently discovered coumarinolignans, are also present in the roots of this model plant (Rohde *et al.*, 2004; Bednarek *et al.*, 2005; Kai *et al.*, 2006; Kai *et al.*, 2008; Schmid *et al.*, 2014; Schmidt *et al.*, 2014; Ziegler *et al.*, 2016; Sisó-Terraza *et al.*, 2016; Ziegler *et al.*, 2017). In a previous study, we reported a significant natural variation in scopoletin and scopolin accumulation between various Arabidopsis accessions (Siwinska *et al.*, 2014). It is interesting that Strategy I plants differ considerably between plant species and genotypes in their tolerance of Fe deficiency. In Arabidopsis, an accumulation of various coumarins, in particular scopoletin and fraxetin, together with their corresponding glycosides, was shown to be highly induced in response to Fe-limited conditions (Fourcroy *et al.*, 2014; Schmid *et al.*, 2014; Schmidt *et al.*, 2014). To date, no enzymes involved in the last step of fraxetin biosynthesis have been identified.

The biosynthesis of coumarins and their accumulation occur in response to Fe deficiency, but the exact mechanisms of action underlying these processes have remained largely unknown. To better understand these mechanisms, we have selected and functionally characterized an enzyme with a previously unknown biological role, encoded by the *At3g12900* gene, which shares significant homologies with the F6ʹH1 and F6ʹH2 enzymes described by Kai *et al.* (2008) as being involved in the synthesis of scopoletin, and which is highlighted in the literature as being strongly iron-responsive (Lan *et al.*, 2011; Rodríguez-Celma *et al.*, 2013*b*; Mai *et al.*, 2015; Mai and Bauer, 2016). This led us to identify the At3g12900 oxidoreductase as a scopoletin 8-hydroxylase (S8H) involved in the biosynthesis of fraxetin, which is associated with the regulation of Fe homeostasis in Arabidopsis.

## Materials and methods

### Plant material

The *Arabidopsis thaliana* Col-0 accession was used as the wild type (WT). The *s8h-1* (SM_3.27151) and *s8h-2* (SM_3.23443) T-DNA insertional mutant lines (Supplementary Fig. S1 at *JXB* online) were identified in the SM (Suppressor-mutator) collection (http://signal.salk.edu/; Tissier *et al.*, 1999) of single-copy dSpm insertions (in the Col-0 background). Seeds of both *s8h* lines are available at the Nottingham Arabidopsis Stock Centre (http://arabidopsis.info/). Genotyping of *s8h-1* and *s8h-2* was done using protocols and primers described in Supplementary Table S1. Selected *s8h-1* homozygous mutants were tested for *S8H* gene expression by quantitative real-time reverse transcription–PCR (qRT–PCR) (Supplementary Fig. S2, Supplementary Table S2). A lack of *S8H* transcript in the *s8h-2* mutant background was confirmed by RT–PCR using primers shown in Supplementary Table S2. *Nicotiana benthamiana* seeds were kindly gifted by Dr Etienne Herbach (INRA, Colmar, France).

### Growth conditions

#### Hydroponic cultures

Plants were grown in two types of modified Heeg solution (Heeg *et al.* 2008), described in detail in Supplementary Table S3. The first solution (10× Heeg) was prepared as previously described (Ihnatowicz *et al.*, 2014); a second solution (1× Heeg) contained 10-fold lower concentrations of microelements (except for Fe^2+^). Arabidopsis seeds were surface sterilized by soaking in 70% ethanol for 2 min and subsequently kept in 5% calcium hypochlorite solution for 8 min. Afterwards, seeds were rinsed three times in autoclaved deionized water. The surface-sterilized seeds were sown on tip-cut 0.65% agar-filled tubes or on microcentrifuge tube lids filled with solidified Heeg medium, which were placed into tip boxes with control hydroponic solution (40 µM Fe^2+^). After a few days’ stratification at 4 °C, boxes with plants were kept in a controlled environment (16 h light at 22 °C/~100 μmol m^−2^ s^−1^ and 8 h dark at 20 °C). Approximately 3 weeks later, plants were transferred either to a freshly made control solution or to Fe-deficient (10 µM Fe^2+^) or Fe-depleted (0 µM Fe^2+^) solutions. In the first set of experiments hydroponic solutions were fully changed once per week, while in the second set of experiments boxes containing old nutrient solution were replenished by the addition of fresh medium.

#### In vitro cultures on plates

Surface-sterilized seeds were sown on Petri dishes containing different homemade Murashige and Skoog (MS) media: (i) half-strength MS medium (0.5 MS) with macroelements at half strength and microelements/vitamins at full strength, and (ii) one-quarter-strength MS medium (0.25 MS) with macroelements at one-quarter strength and microelements/vitamins at half strength. Both types of media contained 1% sucrose, 0.8% agar supplemented with 4 mg l^–1^ glycine, 200 mg l^–1^ myo-inositol, 1 mg l^–1^ thiamine hydrochloride, 0.5 mg l^–1^ pyridoxine hydrochloride, and 0.5 mg l^–1^ nicotinic acid. For stratification, plates were kept in the dark at 4 °C for 72 h and then placed under defined growth conditions [16 h light (~70 μmol m^−2^ s^−1^) at 22 °C and 8 h dark at 20 °C].

#### In vitro liquid cultures

Ten-day-old seedlings from agar plates were transferred into a 250 ml wide-mouth Erlenmeyer flask containing 20 ml sterile 0.25 MS liquid medium. Plants grown in liquid cultures were incubated on rotary platform shakers at 120 rpm. Ten days after transfer, 20 ml of fresh medium was added to each flask. Plants were harvested 18 days after transfer (on the 29th day of culture); leaves and roots were frozen separately in liquid nitrogen and stored at −80 °C.

Two weeks after germination, *N. benthamiana* seeds were transplanted and cultured independently for 3 additional weeks in plant growth chambers under a photoperiod of 16 h light (120 μmol m^−2^ s^−1^) at 24 °C and 8 h dark at 22 °C with 70% humidity.

### Chemicals

The following acids were used: ferulic (Aldrich), coumaric (Sigma), cinnamic (Sigma), and caffeic (Fluka). Coumarins used were: coumarin (Sigma), daphnetin (Sigma), esculetin (Sigma), esculin (Sigma), fraxetin (Extrasynthèse), fraxin (Extrasynthèse), isoscopoletin (Extrasynthèse), limetin (Herboreal), scoparon (Herboreal), 6-methoxycoumarin (Apin Chemicals), 7-methoxycoumarin (Herboreal), scopoletin (Herboreal), scopolin (Aktin Chemicals Inc.), umbelliferone (Extrasynthèse), skimmin (Aktin Chemicals Inc.), and 4-methylumbelliferon (Sigma). The CoA thiol esters of the cinnamates (cinnamoyl-CoA, *p*-coumaroyl-CoA, caffeoyl-CoA, and feruloyl-CoA) were enzymatically synthesized as described by Vialart *et al.* (2012). *P*-coumarate and coenzyme A (CoA) were purchased from Sigma-Aldrich. Kanamycin, chloramphenicol, and isopropyl-β-d-thio-galactopyrannoside (IPTG) were purchased from Duchefa.

### Construction of binary vector and *Agrobacterium tumefaciens* strains

The amplified *S8H* ORF (details of the PCR are provided in Supplementary Table S4) was first cloned into the pCR8 plasmid using the pCR^®^8/GW/TOPO^®^ TA cloning kit (Invitrogen) (Vialart *et al.*, 2012) using Gateway technology. The recombinant pBIN-GW-S8H vector was then introduced into the LBA4404 *A. tumefaciens* strain and used together with the C5851 *A. tumefaciens* strain containing pBIN61-P19 (Voinnet *et al.*, 2003), provided by D. Baulcombe (Department of Plant Science, University of Cambridge, UK) for transient expression in *N. benthamiana* leaves.

### Construction of pET28a+ expression vector and *E. coli* Rosetta 2 strain

The ORF of *S8H* was amplified by PCR (5ʹ primer: 5ʹ-*GGATCC*GGTATCAATTTCGAGGACCAAAC-3ʹ and 3ʹ primer: 5ʹ-*CTCGAG*CTCGGCACGTGCGAAGTCGAG-3ʹ) and cloned between the *Bam*HI and *Xho*I sites of pET28a+. The recombinant plasmid was introduced into the competent *E. coli* Rosetta 2 (Novagen) strain by heat shock.

### Heterologous expression and purification of S8H

The *E. coli* Rosetta 2 strain transformed with pET28a+-S8H was cultured at 37 °C overnight in 10 ml lysogeny broth (LB) (0.5% yeast extract, 1% tryptone, and 1 % NaCl; Sambrook and Russell, 2001) supplemented with 100 mg l^–1^ kanamycin and 33 mg l^–1^ chloramphenicol. A 2 ml pre-culture was transferred to 1 litre of fresh LB containing 100 mg l^–1^ kanamycin and 33 mg l^–1^ chloramphenicol. The induction of S8H expression was adapted from the protocol developed by Oganesyan *et al.* (2007). Transformed cells were cultured at 37 °C until OD_600nm_ reached 0.6. Salt stress with 0.5 M NaCl and heat stress at 47 °C were applied for 1 h in the presence of 2 mM betaine. The temperature was then set at 20 °C for 1 h, and finally the expression of S8H was initiated by adding 1 mM IPTG. Cells were harvested after 14 h by centrifugation for 20 min at 4000 *g* at 4 °C, and the pellet was resuspended in 4 ml potassium phosphate buffer, pH 8.0, with 10 mM imidazol. The cell suspension was sonicated (Bandelin SONOPLUS apparatus) for five periods of 20 s with an interval of 30 s at 50–60% power, and subsequently centrifuged for 20 min at 10000 *g* at 4 °C. The purification of soluble recombinant His-tagged proteins was done using TALON Metal Affinity Resin (TAKARA) as described by the supplier. The purified proteins were eluted with 0.1 M potassium phosphate buffer and 200 mM imidazol solution (pH 8.0).

### Measurement of enzymatic activities

Enzymatic activities were assessed immediately after the purification of S8H. The protocol was adapted from Kai *et al.* (2008). The reaction was done in a 200 µl volume at a saturating concentration of FeSO_4_ (0.5 mM), α-ketoglutarate (5 mM), sodium ascorbate (5 mM) in 0.1 M potassium phosphate buffer at optimal pH (7.0), with 200 µM substrate and 2.6 µg of purified enzyme. Reaction mixtures were incubated for 10 min at an optimal temperature of 31.5 °C. Reaction mixtures with CoA esters were additionally incubated at the end of the reaction with 20 µl of 5 M NaOH for 20 min at 37 °C to hydrolyse the ester bond, and subsequently with 20 µl of acetic acid in order to close the lactone ring. In samples with acids and coumarins as substrates, the reaction was stopped by the addition of 2 µl of trifluoroacetic acid and incubation at 20 °C for 20 min. Reaction mixtures were then centrifuged for 30 min at 10000 *g*; the supernatant was recovered, filtered (0.22 µm), and analysed by high-performance liquid chromatography (HPLC).

### HPLC

Chromatographic separation was performed using an HPLC Shimadzu system with diode array detector/UV detector. The injected volume was 50 µl. Samples were separated on an Interchim C18 Lichrospher OD2 (250 × 4.0 mm, 5 µm) column with the flow speed set to 0.8 ml min^–1^. Separation was performed by elution with the following programme (A) H_2_O and 0.1% acetic acid (B) methanol and 0.1% acetic acid: 0–35 min gradient 10–70% B, 35–36 min 70–99% B, 36–39 min 99–99% B, isocratic elution and column regeneration. The wavelength was set at 338 nm to monitor the formation of fraxetin.

### UHPLC and LTQ LC-MS analysis

Sample analysis was performed on a Nexera UHPLC system (Shimadzu Corp., Kyoto, Japan) coupled with an LCMS 2020 mass spectrometer (Shimadzu). The chromatographic column was a C18 reverse phase (Zorbax Eclipse Plus, Agilent Technologies, Santa Clara, CA, USA) 150 × 2.1 mm 1.8 µm. Elution of the compounds was performed as described by Dugrand *et al.* (2013). To confirm the *in vitro* metabolization of scopoletin into fraxetin, separation was conducted on an Ultimate 3000 chromatographic chain coupled with an LTQ-XL mass spectrometer (Thermo Electron Corporation, Waltham, MA, USA), as described by Karamat *et al.* (2012).

### Heterologous expression in *N. benthamiana* leaves

The protocol for heterologous expression was adapted from Voinnet *et al.* (2003). Several freshly spread colonies of *A. tumefaciens* [LBA4040 (pBIN-F6ʹH2), LBA4040 (pBIN-S8H), C5851 (pBIN61-P10)] were inoculated into 40 ml LB medium containing the antibiotic corresponding to the resistance provided by the plasmids and incubated at 28 °C overnight. The cultures were centrifuged for 10 min at 4000 *g* and the pellet was resuspended in 20 ml sterile deionized water. This step was repeated three times in order to remove all traces of antibiotics. *N. benthamiana* leaves were infiltrated with a suspension of recombinant strains at an OD_600_ of 0.4 for pBIN61-P19 bacteria and 0.2 for pBIN-S8H or pBIN-F6ʹH2 bacteria. The infiltrated plants were stored in a growth chamber for 96 h. In parallel, for each experiment, *N. benthamiana* were infiltrated with LBA4404 (pBIN-GFP) as a positive control. Expression of green fluorescent protein was assessed by examination under a binocular microscope.

### Extraction of polyphenols from *N. benthamiana* leaves

Freshly harvested infiltrated *N. benthamiana* leaves were crushed in liquid nitrogen and a 100 mg sample was mixed with 800 µl 80% methanol. The solution was vigorously mixed for 30 s and then centrifuged for 30 min at 10000 *g*. The supernatant was transferred to a fresh tube and the pellet was submitted to a second extraction with 800 µl 80% methanol. Both supernatants were pooled and vacuum dried. The pellet was resuspended in 100 µl MeOH/H_2_O 80%/20% v/v and analysed by UHPLC.

### Preparation of methanol extracts from Arabidopsis roots

Arabidopsis roots were frozen in liquid nitrogen and ground with a pestle and mortar. A 50 mg sample of plant tissue was mixed in 500 µl of 80% methanol supplemented with 2.5 µM 4-methylumbelliferone as an internal standard. Samples were sonicated for 10 min and centrifuged for 10 min at 10000 *g*. Supernatants were transferred to fresh tubes, vacuum dried, resuspended in 100 µl MeOH/H_2_O 80%/20% v/v, and analysed by UHPLC.

### Extraction of root exudates from nutrient solutions

Nutrient solutions from Arabidopsis *in vitro* cultures were collected 18 days after the onset of Fe treatments. Phenolic compounds were retained in a BAKERBOND™ C18 column (J. T. Baker Chemical Co., Phillipsburg, NJ, USA), eluted from the cartridge with 3 ml of 100% methanol, and dried in a centrifugal evaporator. Dry extracts were stored at –20 °C until further analysis.

### Trace element analysis

Arabidopsis roots were first lyophilized and subsequently milled before microwave (MARS)-assisted digestion in concentrated nitric acid (Aristar). Metal concentrations were determined by inductively coupled plasma-mass spectrometry (ICP-MS) (Thermo iCAP Q, Bremen, Germany).

### Measurement of chlorophyll

Chlorophyll extraction with acetone was done according to the method of Porra *et al.* (1989). The absorbances of the diluted supernatants were measured at 750.0, 663.6, and 646.6 nm. After measurement, a formula was used to convert absorbance measurements to mg of chlorophyll (Porra *et al.*, 1989).

### Statistical analysis

All treatments included at least three biological replicates. Data processing and statistical analyses (pairwise comparisons using *t*-tests, except for trace element analysis) were done using Excel 2010 (Microsoft). Statistical significance of differences observed in trace element analysis were analyzed by R version 3.3.2 (2016-10-31) (R Core Team 2014, https://www.R-project.org/) with the use of the agricolae package.

## Results

### Phylogenetic analysis of genes encoding Fe^2+^- and 2OG-dependent dioxygenases from the Arabidopsis genome

**Fig. 1.**
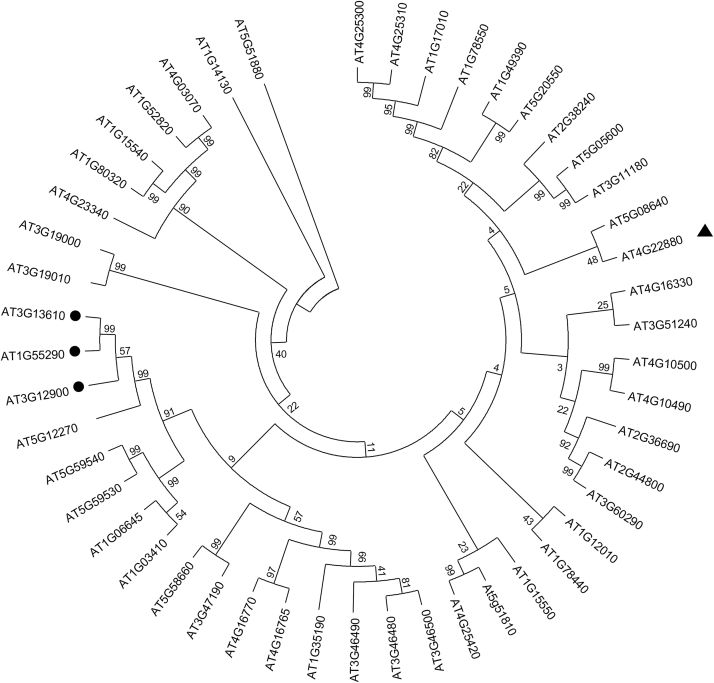
Nucleic acid sequence-based tree. The phylogenetic analysis was performed on available nucleic acid sequences of genes encoding 2-oxoglutarate (2OG)- and ferrous iron Fe^2+^-dependent dioxygenase (2OGD) from the Arabidopsis genome. The phylogenetic tree was made using MEGA 6 software. Black circles indicate genes (*At3g13610* and *At1g55290*) encoding enzymes involved in scopoletin biosynthesis (F6ʹH1 and F6ʹH2 respectively), and their closest homologue based on the nucleic acid sequences, a gene (*At3g12900*) encoding a dioxygenase of unknown biological function (characterized in this study as S8H). The black triangle indicates a gene (*At4g22880*) encoding the anthocyanidin synthase (ANS) enzyme. Nucleic sequences downloaded from TAIR were selected based on the presence of sequences coding for Fe-binding (His-X-Asp-X-His) and 2OG-binding (Arg-X-Ser) motifs (Wilmouth *et al.*, 2002).

More than 100 genes encoding enzymes sharing sequence homology with dioxygenases were identified in the Arabidopsis genome (Kawai *et al.*, 2014). Almost half of them display characteristic amino acid sequence motifs involved in binding cofactors such as Fe^2+^ and 2OG (His-X-Asp-X-His and Arg-X-Ser, respectively; Wilmouth *et al.*, 2002). We performed phylogenetic analysis based on the nucleic acid sequences of putative genes encoding dioxygenases collected from The Arabidopsis Information Resource (TAIR; http://arabidopsis.org/). This analysis indicated that two genes involved in scopoletin biosynthesis (*At3g13610* and *At1g55290*, encoding F6ʹH1 and F6ʹH2, respectively) were clustered together, and identified *At3g12900*, a gene of unknown function, as sharing the highest homology with both scopoletin synthases (Fig. 1).

The predicted amino acid sequences of the three genes were compared to that of a leucoanthocyanidin dioxygenase, named anthocyanidin synthase (ANS) and encoded by *At4g22880*, which catalyses the conversion of leucoanthocyanidins into anthocyanidins (Heller and Forkmann, 1986) and has the highest sequence identity to F6ʹH1 among enzymes whose crystallographic structure is known (Wilmouth *et al.*, 2002). The Fe^2+^ binding site (His 235, His 293, Asp 237 in F6ʹH1) and the 2OG binding site (Arg 303 and Ser 305 in F6ʹH1) are highly conserved in all proteins (Supplementary Fig. S3). In contrast, the amino acid residues responsible for substrate binding differ among the enzymes, which is consistent with the activities already described for three of them: feruloyl-CoA ester for F6ʹH1 and F6ʹH2 (Kai *et al.*, 2008), and leucoanthocyanidin for ANS (Wilmouth *et al.*, 2002). These alignments highlight a different sequence for At3g12900, which suggests that it has another substrate specificity.

### Determination of *in vitro* substrate specificity and kinetic parameters of enzymatic reaction catalysed by the At3g12900 oxidoreductase

**Fig. 2.**
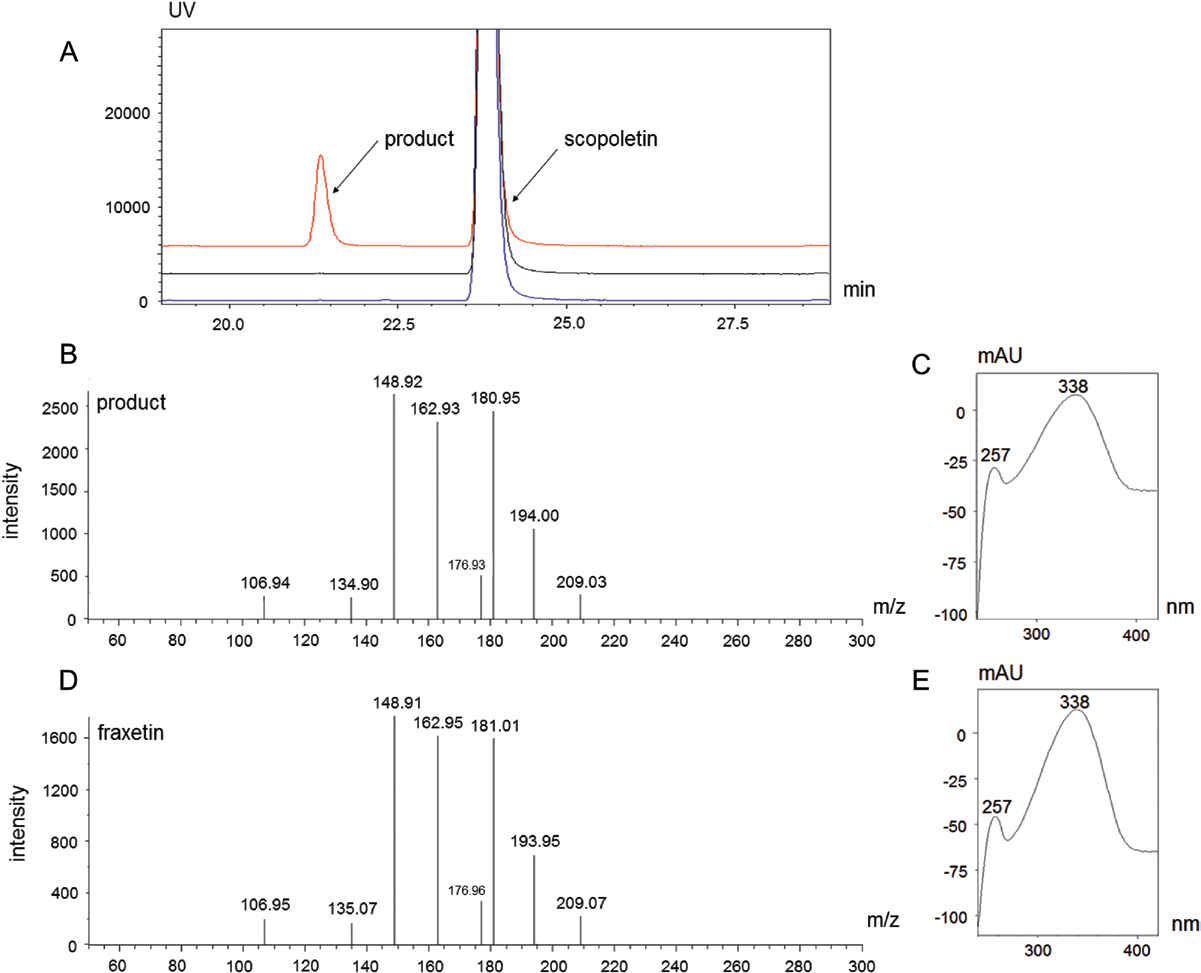
*In vitro* functional characterization of the S8H enzyme. (A) The chromatograms in blue and black are the two negative control reactions. Blue shows the reaction carried out without the addition of iron, and black without the addition of oxoglutarate. The chromatogram in red shows the result of incubation of the S8H enzyme with scopoletin and enzyme cofactors Fe^2+^ and 2OG. (B) MS fragmentation of the additional peak from the chromatogram shown in red in A. (C) UV absorbance spectrum of the additional peak from the chromatogram indicated in red in A. (D) MS fragmentation of a fraxetin standard. (E) UV absorbance spectrum of a fraxetin standard.

**Fig. 3.**
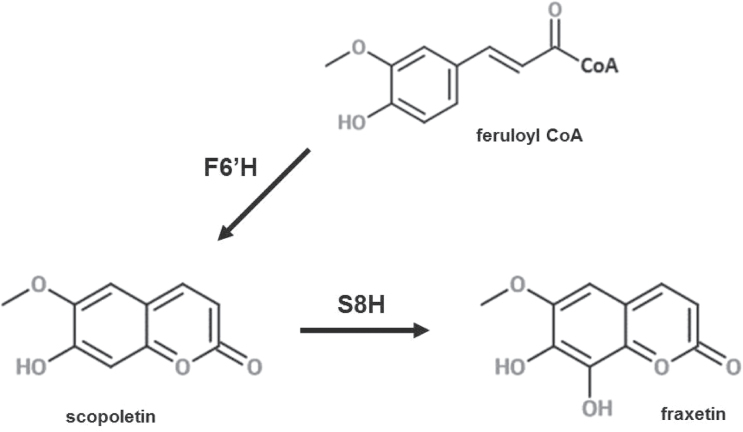
Last step of fraxetin biosynthesis in *Arabidopsis thaliana* catalysed by scopoletin 8-hydroxylase (S8H). The Fe^2+^- and 2-oxoglutarate-dependent dioxygenase (F6ʹH) catalyses the ortho-hydroxylation of feruloyl-CoA before the lactone ring formation to produce scopoletin. Subsequently, S8H catalyses hydroxylation at the C8 position of scopoletin, leading to fraxetin production.

In order to determine the substrate specificity of the At3g12900 enzyme, an At3g12900 6× His-tagged protein was expressed in *E. coli*. An improved protocol was used for purification of the heterologously expressed enzyme to obtain the purified protein fraction (Supplementary Fig. S4). The purified enzyme was incubated in the presence of dioxygenase cofactors at saturating concentrations and 19 various potential substrates belonging to (i) cinnamoyl derivative CoA esters, (ii) cinnamic derivative acids, and (iii) coumarins (Supplementary Table S5). Only scopoletin was converted into a product (Fig. 2A), which was unambiguously identified as fraxetin by its UV absorption spectrum, its molecular mass (Fig. 2A–C), and its MS fragmentation spectrum in comparison to a fraxetin commercial standard (Fig. 2D, E). The At3g12900 protein can therefore be considered to be a scopoletin 8-hydroxylase (S8H) (Fig. 3) that catalyses hydroxylation at the C8 position of scopoletin. The optimal reaction conditions were determined to be pH 8.0 ± 0.1 at 31.5 ± 0.4 °C (Supplementary Fig. S5A, B) and the optimal incubation time was set at 10 min (Supplementary Fig. S6A). Under these experimental conditions, we determined the kinetic characteristics of the enzyme as being *K*_*m*_ 11 ± 2 µM, *K*_cat_ 1.73 ± 0.09 s^–1^ (Supplementary Fig. S7). We also demonstrated that the enzyme efficiency was best in the presence of 50 µM Fe^2+^ (Supplementary Fig. S6B) and that higher concentrations of Fe^2+^ significantly reduced the enzyme activity, leading to decreased fraxetin synthesis.

### *In vivo* activity of scopoletin 8-hydroxylase in *N. benthamiana*

**Fig. 4.**
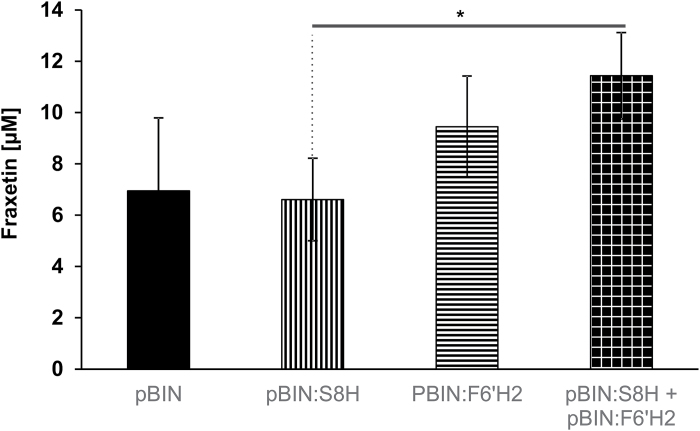
Fraxetin content in *Nicotiana benthamiana* leaves. Transient expression was carried out using *Agrobacterium tumefaciens* transformed with the pBIN empty vector (control), pBIN:S8H, pBIN:F6ʹH2, and simultaneously with vectors encoding F6ʹH2 and S8H. Fraxetin content was quantified using UHPLC. Data are presented as mean ±SD. *P<0.05.

To confirm its function in plant cells, we transiently expressed the S8H enzyme in *N. benthamiana* leaves. Our analysis showed that the presence of this enzyme did not increase the production of fraxetin in comparison to a control infiltration performed with *A. tumefaciens* transformed with an empty vector (Fig. 4). However, when we conducted a double infiltration leading to the overexpression of S8H and F6ʹH2 in tobacco leaves, we detected a significantly higher level of fraxetin in comparison to leaves transiently expressing S8H alone (*P*=0.01) (Fig. 4). This result is consistent with an increase of the scopoletin pool that occurs due to F6ʹH activity, which can be further transformed into fraxetin. It also confirms that At3g12900 functions as a S8H.

### Characterization of independent *s8h* mutant alleles grown in Fe-depleted hydroponic conditions

**Fig. 5.**
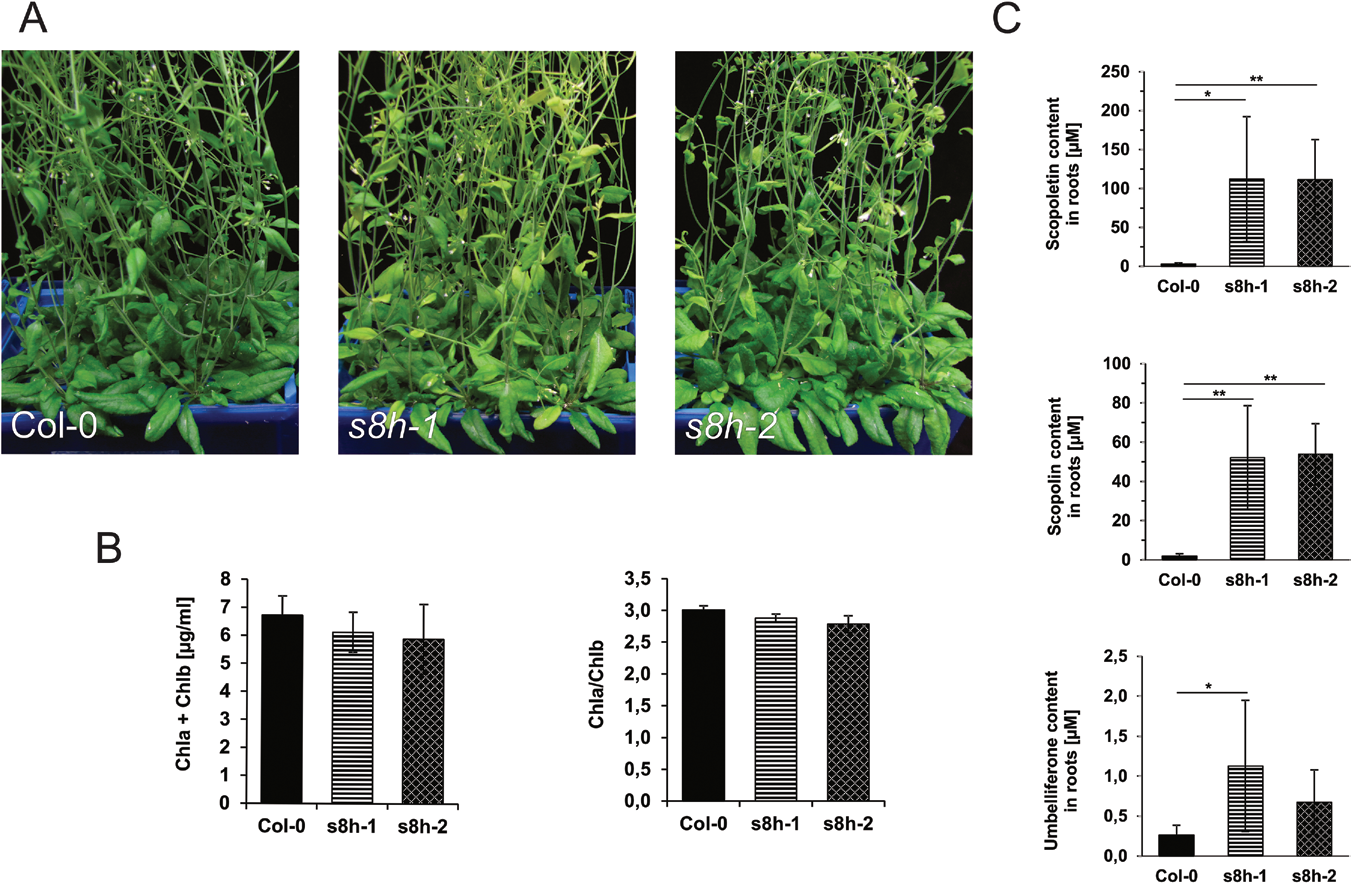
Phenotypic characterization of 6-week-old wild-type (Col-0) plants and two *s8h* T-DNA mutant lines (*s8h-1* and *s8h-2*) grown in Fe-depleted hydroponic solution (0 µM Fe^2+^). After 3 weeks of growth in optimal hydroponic solution (40 µM Fe^2+^), plants were transferred to (A) freshly made Fe-depleted solutions and cultured until the chlorotic phenotype was clearly visible. Hydroponic solutions were fully changed once per week. (B) Chlorophyll (Chl) a+b concentration and a/b ratio of wild-type and mutant plants. (C) Relative levels of scopoletin, scopolin, and umbelliferone accumulated in the plant roots. Metabolite profiling of coumarins was done by UHPLC. The results of one representative experimental replicate are presented. Data are presented as mean ±SD from six biological replicates. *P<0.05, **P<0.01.

To gain insight into the physiological role of S8H, we identified two independent mutant lines with non-functional S8H dioxygenase (*s8h-1* and *s8h-2*) and investigated their behaviour in various types of culture. Because the literature has reported that fraxetin accumulation is induced under conditions of Fe deficiency, we investigated the behaviour of the mutants in Fe-depleted hydroponic solutions. Plants were grown in a control hydroponic solution (40 µM Fe^2+^) for 3 weeks and subsequently transferred to Fe-depleted solution (0 µM Fe^2+^) and cultured for an additional 3 weeks. In Fe-depleted solutions both mutant lines were clearly paler in colour than Col-0 control plants (Fig. 5A). The mutant phenotype was linked with a lower chlorophyll a+b content and chlorophyll a/b ratio (Fig. 5B). Targeted metabolite profiling of methanolic extracts from the roots of plants grown in hydroponic systems showed a highly significant increase in scopolin (*P*<0.01) and scopoletin (*P*<0.01) concentration in roots grown in Fe-depleted conditions for both mutant lines, and a significant increase in umbelliferone accumulation in *s8h-1* compared with Col-0 plants (Fig. 5C).

### Biochemical and ionomic characterization of plants cultivated in liquid cultures with different Fe concentrations

**Fig. 6.**
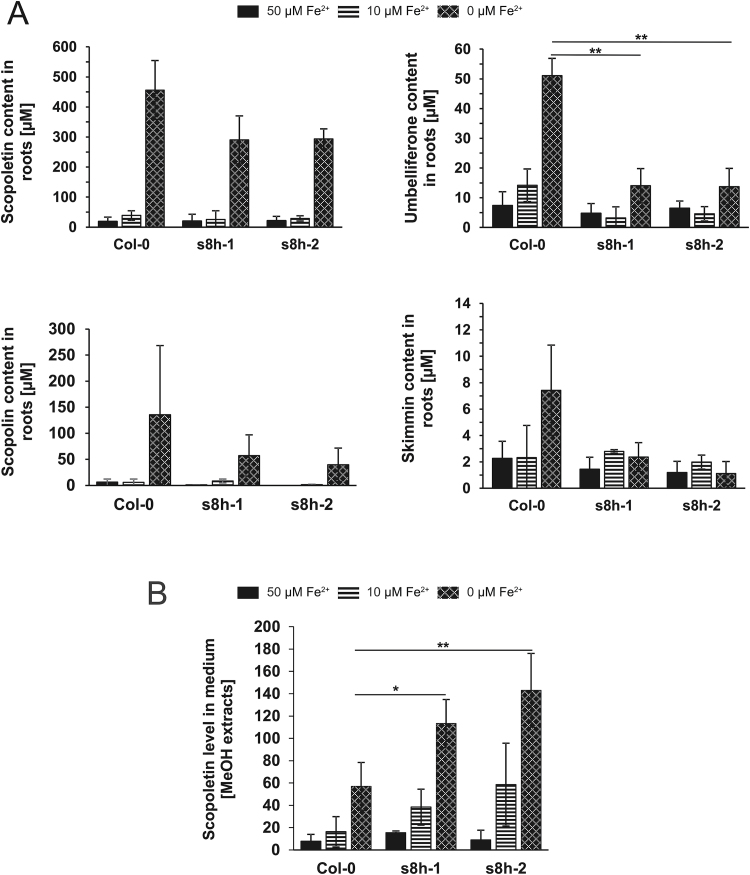
Biochemical characterization of *Arabidopsis thaliana s8h* mutants and Col-0 plants grown *in vitro* in 0.25 MS liquid cultures with various Fe content (0, 10, and 50 µM Fe^2+^). Relative levels of scopoletin, scopolin, umbelliferone and skimmin in the root exudates (A) and scopoletin levels in the methanol root extracts (B) of *s8h* mutants and Col-0 plants. Metabolite profiling of coumarins was done by UHPLC. Data are presented as mean ±SD from three measurements. *P<0.05, **P<0.01.

Since coumarins are mostly stored in roots, all tested genotypes were cultivated *in vitro* in liquid cultures, in order to obtain enough root biomass for analysis of trace elements and media for extraction of root exudates and metabolic profiling. After 3 weeks of growth, no visible phenotypic differences could be observed among WT and mutant plants: all genotypes grown in Fe-deficient medium (10 µM Fe^2+^) were paler and plants grown in Fe-depleted medium (0 µM Fe^2+^) were chlorotic (Supplementary Fig S8). Chlorophyll content was slightly higher in both mutant lines in all media tested, while the chlorophyll a/b ratio was significantly higher in Col-0 plants in control media and slightly higher in Fe-deficient and Fe-depleted media (Supplementary Fig. S9). However, the targeted metabolite profiling of root extracts and root exudates showed that under Fe-depleted conditions both mutant lines accumulated lower levels of various coumarins (scopoletin, scopolin, umbelliferone, and skimmin) (Fig. 6A) but secreted significantly higher amounts of scopoletin in comparison to WT plants (Fig. 6B).

**Fig. 7.**
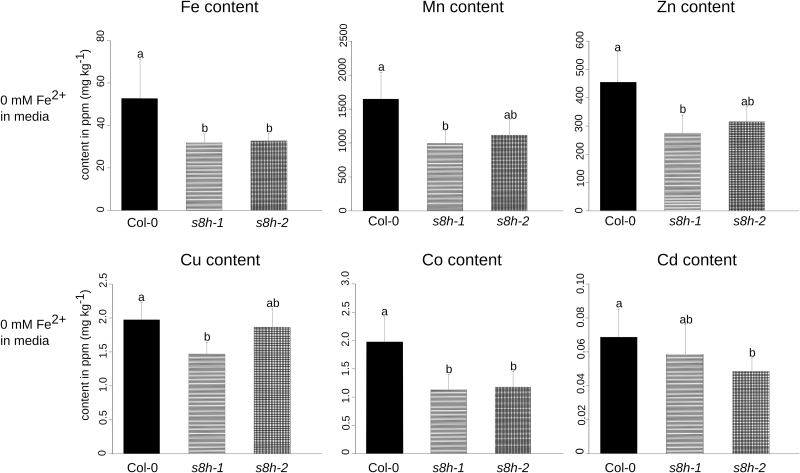
Microelement heavy metal contents of Col-0 plants and *s8h* mutant roots grown *in vitro* for 3 weeks in Fe-depleted (0 µM Fe^2+^) 0.25 MS liquid culture. Microelement concentrations, shown in ppm (mg kg^−1^), were determined by inductively coupled plasma-mass spectrometry (ICP-MS). The results of one representative experimental replicate are presented. Data are presented as mean ±SD from five biological replicates. Values that are significantly different (*P*<0.05) are indicated by different letters above the bars.

The trace element analysis of plants grown in liquid culture media with various Fe levels indicated that, when grown in Fe-depleted medium (0 µM Fe^2+^), the Fe content of both mutant lines (*s8h-1* and *s8h-2*) was significantly lower (*P*<0.05) in comparison to the corresponding control (Col-0) (Fig. 7). The concentration of a range of other microelement heavy metals (Mn, Zn, Cu, Co, and Cd) was also significantly lower in the *s8h* mutants compared with control plants (*P*<0.05) grown in Fe-depleted medium (Fig. 7).

As phosphate (Pi) and Fe deficiency interact with respect to Fe-induced coumarin secretion (Ziegler *et al.*, 2016), and our soil experiments found that Col-0 and *s8h-1* phenotypes were dependent on the P to Fe ratio (Supplementary Fig. S10, Supplementary Table S6), another observation from the trace element analysis is the lower levels of P content in both mutants (significantly lower in the *s8h-1* line, *P*<0.05) in comparison to Col-0 plants under Fe-depleted (0 µM Fe^2+^) conditions (Supplementary Figure S11A). Plants grown in Fe-deficient (10 µM Fe^2+^) and Fe-optimal (50 µM Fe^2+^) media did not show changes in trace element contents (Supplementary Fig. S11A–C), with the exception that there was a significantly higher Mo content in *s8h* mutants grown in Fe-deficient medium (Supplementary Fig. S11C).

### Phenotyping of *s8h* mutants grown on MS plates and in hydroponic solutions with various concentrations of Fe and other microelements

**Fig. 8.**
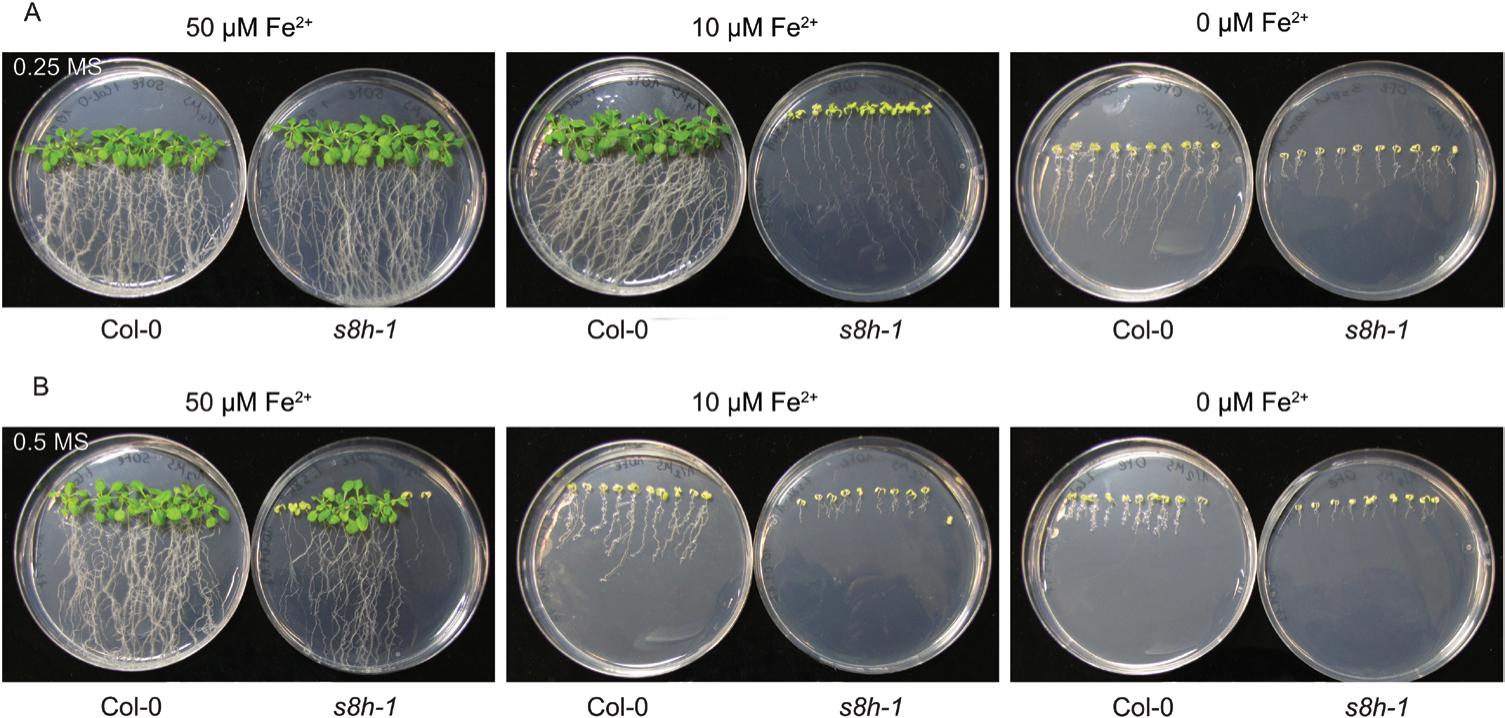
Phenotypic appearance of 3-week-old Col-0 and *s8h-1* plants grown on (A) 0.25 MS and (B) 0.5 MS media. Plants were grown on MS media with various Fe content (0–50 µM Fe^2+^) in plant growth chambers under a photoperiod of 16 h light (~70 μmol m^−2^ s^−1^) at 22 °C and 8 h dark at 20 °C. A second mutant allele (*s8h-2*) showed a very similar response (data not shown).

The impact of trace elements on Arabidopsis growth was further investigated by cultivating the *s8h* mutants and control plants on plates and hydroponic cultures characterized by different concentrations of Fe and other microelements. To observe the growth of both rosettes and roots, the MS plates were placed in a vertical and a horizontal position. The growth of all genotypes was more extensive on 0.25 MS medium than on the corresponding plates with 0.5 MS medium, irrespective of the concentration of Fe (Supplementary Fig. S12). These differences were particularly striking after 3 weeks of cultivation. When grown on 0.25 MS Fe-deficient (10 µM Fe^2+^) medium, the *s8h* mutants showed a strong reduction in fresh weight (Supplementary Fig. S13A), were much paler, and had shorter and fewer lateral roots compared with Col-0 plants (Fig. 8A). On 0.5 MS Fe-deficient medium, both genotypes displayed a significant reduction in shoot and root growth, but *s8h* mutants showed a smaller rosette size than Col-0 plants (Fig. 8B). Under both Fe-depleted conditions (0 µM Fe^2+^), mutant lines and Col-0 plants showed significantly smaller and chlorotic shoots, while roots were markedly shorter (Fig. 8A, B). However, the WT plants grew slightly more. The shoots of *s8h* mutants grown on 0.25 MS Fe-optimal (50 µM Fe^2+^) medium were clearly larger than the WT plants; this was not the case on 0.5 MS medium with optimal Fe content (Fig. 8A, B).

Examination of plants grown on Fe-deficient plates (10 µM Fe^2+^) under UV light showed that both *s8h* mutant lines secreted a higher level of fluorescent compounds into the medium compared with WT plants (Supplementary Fig. S14A); this observation might be related to the significantly higher amounts of scopoletin detected in Fe-deficient liquid culture solution of *s8h* plants (Fig. 6B). This difference in fluorescence was not observed in Fe-optimal conditions (50 µM Fe^2+^), in which the mutant plants seemed to accumulate slightly more fluorescent compounds in their roots compared with WT plants (Supplementary Figure S14B).

Similar to plants grown *in vitro*, plants cultured in hydroponic conditions also showed phenotypic variation in plant responses, which was dependent not only on Fe but also on the concentrations of other micronutrients. Both *s8h* mutants were paler than Col-0 plants when grown in 1× Heeg Fe-depleted (0 µM Fe^2+^) solution, which contains 10-fold lower concentrations of microelements than the other hydroponic solution used (Supplementary Table S3). This phenotype was associated with lower amounts of chlorophylls a and b. Under Fe-deficient (10 µM Fe^2+^) conditions, both mutant lines were much larger than WT plants, with greatly increased fresh weight (Supplementary Figure S15B). Surprisingly, when grown with optimal Fe (40 µM Fe^2+^) but lower amounts of other micronutrients (Supplementary Table S3), all genotypes were significantly smaller but did not show any chlorosis or changes in chlorophyll content (Supplementary Fig. S15A). In contrast, when plants were cultivated in 10× Heeg solution, characterized by higher micronutrient concentrations (Supplementary Table S3), there were no visible differences among *s8h* mutants and WT plants when grown in the presence of different Fe levels (Supplementary Fig. S16). Under optimal and Fe-deficient conditions, all plants displayed a WT-like phenotype with normal pigmentation, but in solutions without Fe all plants were smaller and chlorotic. In these hydroponic experiments, both solutions (1× Heeg and 10× Heeg) were fully exchanged weekly.

There appears to be a crucial role for root-secreted coumarins in the acquisition of Fe (Tsai and Schmidt, 2017). Therefore, the hydroponic culture experiment was repeated without changing the nutrient solution weekly. Instead, the root chambers were replenished by the addition of fresh medium to keep the volume of solution in each culture constant, and to retain potentially secreted coumarins. When grown in 1× Heeg solution, under Fe-depleted conditions (0 µM Fe^2+^) both *s8h* mutants became chlorotic (Supplementary Fig. S17C), as previously observed (Supplementary Fig. S15C). In contrast, the growth of all genotypes was not affected in Fe-optimal solution and under Fe deficiency (40 and 10 µM Fe^2+^, respectively) (Supplementary Fig. S17A, B); this could be due to a higher accumulation of coumarin in hydroponic solutions that were not fully changed. Similar to previously conducted hydroponic experiments, all plants grown in 10× Heeg solution under Fe-deficient and Fe-optimal conditions did not show any sign of chlorosis or growth retardation (Supplementary Figure S18A, B). Under Fe-depleted conditions, all genotypes were only slightly smaller compared with their growth optimal conditions, and they had normally pigmented leaves with no changes in chlorophyll content (Supplementary Figure S18C); only the shoots of the mutant lines seemed to be slightly brighter in colour.

## Discussion

In the Arabidopsis genome there are more than 100 genes encoding enzymes sharing homologies with dioxygenases. The 2OGD family is the second largest enzyme family in plants; its members are involved in various oxygenation/hydroxylation reactions (Kawai *et al.*, 2014), including the biosynthesis of coumarins, which are important compounds contributing to the adaptation of plants to biotic and abiotic stresses (Kai *et al.*, 2008; Vialart *et al.*, 2012; Matsumoto *et al.*, 2012; Rodríguez-Celma *et al.*, 2013*a*). Among the most common stress factors leading to plant growth disorders and chlorosis are a deficiency or excess of micronutrients, which can result in various physiological disorders (Marschner, 2012). Maintaining nutrient homeostasis in cells is crucial for the proper functioning of plants, and the mechanisms governing mineral uptake and transport must be strictly controlled.

Plants use different strategies to compensate for Fe limitation. Recently, it was shown that one of the Arabidopsis Fe^2+^- and 2OG-dependent dioxygenases, the scopoletin synthase F6ʹH1 (Kai *et al.*, 2008), is required for the biosynthesis of the Fe^3+^-chelating coumarin esculetin, which is released into the rhizosphere as part of Fe uptake by Strategy I plants (Schmid *et al.*, 2014).

Here, we have analysed a strongly Fe-responsive gene of previously unknown function, *At3g12900*, which shares high homology with the Fe^2+^- and 2OG-dependent dioxygenase family, and revealed its possible contribution to Fe homeostasis in plants. The *At3g12900* gene was selected on the basis of its high homology to the previously described F6ʹH1 dioxygenase ,which has been shown to play a crucial role in Fe acquisition under alkaline soil conditions (Schmid *et al.*, 2014), and the literature data on plant responses to Fe deficiency at the transcriptome and proteome level (Lan *et al.*, 2011; Rodríguez-Celma *et al.*, 2013*b*; Fourcroy *et al.*, 2014; Schmidt *et al.*, 2014). Similar to F6ʹH1, the protein encoded by *At3g12900* accumulates by several-fold in Fe-deficient roots in comparison to Fe-sufficient roots (Lan *et al.*, 2011). Analysis of large microarray datasets found that both genes (*At3g12900* and *At3g13610*, encoding F6ʹH1) were positively correlated with genes actively involved in the response to Fe deficiency (Vigani *et al.*, 2013) such as *IRT1*, *FRO2*, *CYP82C4* (Murgia *et al.*, 2011), and *Ferroportin/Iron-Regulated* (*IREG2*) (Morrissey *et al.*, 2009), and a gene encoding metal tolerance protein (*MTP3*; Arrivault *et al.*, 2006). Moreover, according to the expression data available at TAIR, *At3g12900* is expressed specifically in the roots. The root tissue is the site of coumarin accumulation induced in response to various environmental stresses, including Fe limitation (Schmid *et al.*, 2014).

We determined the substrate specificity of the enzyme encoded by *At3g12900* to test its possible involvement in coumarin biosynthesis as an important part of the Fe uptake strategy in Arabidopsis. An *in vitro* enzymatic activity assay revealed that this enzyme is involved in the conversion of scopoletin into fraxetin via hydroxylation at the C8 position; consequently, the enzyme was named S8H. The Michaelis constant (*K*_m_=11 µM) determined in our *in vitro* experiments was similar to those reported for the biosynthetic enzymes of specialized metabolism (Kai *et al.*, 2008; Vialart *et al.*, 2012). These results, taken together, suggest that the hydroxylation of scopoletin (and, as an effect, the synthesis of fraxetin) is the main activity of the S8H enzyme. The experiments performed with *in vitro*-produced enzymes also provided evidence that S8H activity was dependent on the concentration of Fe^2+^.

Transient expression of the S8H protein in *N. benthamiana* and a subsequent metabolic analysis done on the infiltrated leaves further confirmed the results of the *in vitro* assay. A simultaneous transformation with the *S8H* and *F6ʹH2* heterologous genes in tobacco leaves resulted in a significantly higher accumulation of fraxetin; the concentration of fraxetin was intermediate when *F6ʹH2* alone was expressed, and much lower when empty vector or *S8H* alone were expressed. The latter finding could be explained by the fact that tobacco plants do not synthesize scopoletin in a constitutive manner, but do synthesize fraxetin. The overproduction of F6ʹH2 therefore induces the synthesis of scopoletin from feruloyl-CoA, which is naturally present in tobacco leaves (Kai *et al.*, 2008), thus providing the substrate for the reaction catalysed by S8H, resulting in a significantly higher fraxetin content.

To better understand the link between Fe homeostasis in plants and the biosynthesis of fraxetin, and consequently to show that the *in vitro* enzyme activity of S8H is relevant *in vivo,* we performed a detailed phenotypic characterization of Col-0 plants and two independent *s8h* mutant lines grown under different Fe regimes using various types of culture. Plants were grown in hydroponic solution, soil mixes, *in vitro* liquid cultures, and on MS plates with various concentrations of Fe and other micronutrients. Our results clearly showed that the *s8h* plants, which carry mutated S8H alleles, are strongly affected by Fe-deficient conditions. Targeted metabolite profiling of *s8h* mutants demonstrated that coumarin profiles are significantly modified in mutant roots grown in Fe-depleted conditions. We detected higher concentrations of scopoletin in exudates from *s8h* mutant roots grown in liquid cultures. This finding was associated with lower levels of various coumarins and lower Fe content in mutant roots as compared with WT plants. Furthermore, the rosettes of *s8h* plants grown in 1× Heeg Fe-depleted hydroponic solution were clearly paler than those of Col-0 plants, and these changes were associated with striking changes in the metabolite profiles of coumarins in mutant roots. In comparison to Col-0 plants, under the 1× Heeg Fe-depleted condition we detected a significantly higher accumulation of scopoletin and scopolin in *s8h* roots that would suggest the inhibition of scopoletin-hydroxylation-dependent synthesis of fraxetin in mutant tissues. It is most likely that the *in vitro* culture conditions in a small volume of liquid solution favour the increased secretion of exudates, and therefore we observed lower levels of various coumarins in *s8h* roots grown in liquid cultures linked to a significantly higher scopoletin content in exudates of mutant roots. The phenotypic differences between WT and mutant genotypes were also apparent on MS plates containing Fe-deficient medium, on which the *s8h* mutants showed chlorosis, significant growth retardation, and secreted an increased level of fluorescent compounds compared with WT plants.

Taking into account the results of soil experiments in which plants were cultured in soil mixtures with different chemical compositions, and the fact that *s8h-1* mutants were larger than Col-0 plants grown in a soil mixture characterized by a relatively low level of P and high level of Fe, it will be also interesting to test the growth and metabolic profiles of roots and root exudes of *s8h* mutants grown in hydroponic solutions with various availability of P. This would be particularly interesting in the light of recent reports on the common and antagonistic regulatory pathways between phosphate (Pi) and Fe deficiency-induced coumarin secretion (Ziegler *et al.*, 2016).

Another area for further investigation is linked to the ICP-MS results indicating that, a range of other metals (Mn, Zn, Cu, Co, and Cd) were also significantly decreased in the *s8h* mutants. This is also linked to the phenotypic variation in plant growth that we observed on MS plates and in hydroponic cultures, which was dependent not only on Fe but also on the concentration of other micronutrients. This finding could be explained by the well-known phenomenon of interdependence of individual micronutrients in relation to each other (Ihnatowicz *et al.* 2014), and the fact that various metals interfere with Fe-deficiency responses (Lešková *et al.* 2017). Nevertheless, our work indicates that to get a broad overview of plant responses to nutrient deficiencies and to better understand the physiological role of the genes/enzymes involved, one needs to consider using various types of cultures, solution, and media in experimental procedures.

Among the numerous targeted metabolite profiling experiments conducted in this study, we repeatedly obtained results clearly showing significantly changed coumarin profiles in both *s8h* mutant lines studied. We observed a significantly higher content of scopoletin in the root tissue in hydroponic experiments and in the root exudates in liquid cultures. Scopoletin is a substrate for the reaction catalysed by S8H. The lack of S8H in the *s8h* mutant background could lead to the accumulation of higher levels of scopoletin and its corresponding glycoside, scopolin.

At the moment, we have no explanation for the fact that only small amounts of fraxetin were detected in some of the Col-0 samples under Fe-deficiency conditions (data not shown). It should also be noted that unexpectedly, in one experimental replicate, we detected relatively high amounts of fraxin and a low level of fraxetin in the *s8h-1* mutant background, as well as low amounts of fraxin in some other Col-0 and *s8h* replicates. It cannot be excluded that these observations could be explained by the presence of another enzyme involved in fraxetin biosynthesis or an alternative metabolic pathway being induced in the *s8h* mutant background. The fraxetin synthesized in Col-0 plants could also be further demethylated to 6,7,8-trihydroxycoumarin, with a beneficial effect for plants under Fe-deficient conditions; alternatively, fraxetin could be directly involved in increasing Fe availability. These possibilities cannot be excluded, taking into account the catechol-type structure of fraxetin, and so they need to be further investigated. As reported by Schmid *et al.* (2014), the Fe-deficient chlorotic phenotype of *f6ʹh1* seedlings grown under low Fe availability could be reversed by exogenous application of esculetin, esculin, and scopoletin. In parallel, the result of *in vitro* assay showed that only esculetin was able to chelate and mobilize Fe^3+^ (Schmid *et al.*, 2014). This suggests that compounds bearing an ortho-catechol moiety, such as esculetin and fraxetin, may be involved in the secretion of coumarin for Fe acquisition. The beneficial effect of exogenous application of scopoletin on reversing the chlorotic phenotype of *f6ʹh1* demonstrated by Schmid *et al.* (2014, could be due to the activity of the S8H enzyme catalysing the hydroxylation of scopoletin, leading to fraxetin formation. Whatever mechanisms underlie such plant responses, given the results of *in vitro* enzyme activity assays and the observation of significant changes in the metabolite profiles of coumarin metabolism in *s8h* mutants grown in Fe-depleted conditions, it seems evident that the At3g12900 oxidoreductase is an S8H involved in fraxetin biosynthesis.

The present results indicate that At3g12900 is an important candidate for having a key role in Fe acquisition by plants. Fraxetin and other root-released phenolic compounds, mainly esculetin deriving from scopoletin and scopolin, have been suggested to be Fe chelators or Fe uptake facilitators in Arabidopsis (Schmid *et al.*, 2014; Fourcroy *et al.*, 2014; Schmidt *et al.*, 2014; Brumbarova *et al.*, 2015), and esculetin and fraxetin or fraxetin-derived compounds are possibly involved in the transport of Fe ions into the plant cell under Fe-deficient conditions. The precise physiological function of the phenolic compounds synthesized by plants under Fe-deficiency stress and the possible mechanism underlying plant responses to Fe limitation under calcareous conditions remain unknown (Schmid *et al.*, 2014). Elucidating the biological role of the S8H enzyme involved in coumarin biosynthesis is a prerequisite to understanding the function of fraxetin in Fe acquisition.

## Supplementary data

Supplementary data are available at *JXB* online.

Fig. S1. Schematic representation of independent T-DNA mutant lines for the *At3g12900* gene.

Fig. S2. Lack of *S8H* transcript in the *s8h-1* T-DNA mutant line confirmed by qRT-PCR.

Fig. S3. Multiple-sequence alignment of amino acid sequences of F6ʹH1, F6ʹH2, S8H and ANS enzymes.

Fig. S4. Results of recombinant His-tagged S8H protein purification.

Fig. S5. The optimal pH and temperature for *in vitro* activity of S8H.

Fig. S6. Characterization of *Arabidopsis thaliana* S8H enzyme activity.

Fig. S7. Kinetic parameters *V*_max_ and *K*_m_ of S8H.

Fig. S8. Phenotypic appearance of Col-0 and *s8h* plants grown *in vitro* in liquid cultures with various levels of Fe.

Fig. S9. Chlorophyll content and chlorophyll a/b ratio of Col-0 and *s8h* plants grown *in vitro* in liquid cultures with various levels of Fe.

Fig. S10. Phenotyping and biochemical characterization of Col-0 and *s8h-1* plants grown in soil mixes with different Fe availability.

Fig. S11. Trace element content of Col-0 and *s8h* plant roots grown *in vitro* in Fe-depleted liquid culture.

Fig. S12. Phenotypic appearance of 2-week-old Col-0 and s*8h-1* plants grown on 0.25 MS and 0.5 MS media.

Fig. S13. Fresh weight of 3-week-old Col-0 and *s8h* plants grown *in vitro* on 0.25 MS and 0.5 MS media.

Fig. S14. Phenotypic appearance of Col-0 and s*8h* plants observed under UV light.

Fig. S15. Phenotypic characterization of Col-0 and *s8h* plants grown in 1× Heeg hydroponic solution (fully changed once per week) with 0–40 µM Fe^2+^ content.

Fig. S16. Phenotypic characterization of Col-0 and *s8h* plants grown in 10× Heeg hydroponic solution (fully changed once per week) with 0–40 µM Fe^2+^ content.

Fig. S17. Phenotypic characterization of Col-0 and *s8h* plants grown in 1× Heeg hydroponic solution (refilled with a fresh medium) with 0–40 µM Fe^2+^ content.

Fig. S18. Phenotypic characterization of Col-0 and *s8h* plants grown in 10× Heeg hydroponic solution (refilled with a fresh medium) with 0–40 µM Fe^2+^ content.

Table S1. Genotyping of *s8h* mutant lines.

Table S2. Lack of *S8H* transcript in the *s8h* mutant backgrounds.

Table S3. Modified Heeg solutions used in hydroponic cultures.

Table S4. PCR amplification of the *S8H* ORF.

Table S5. Compounds used in the analysis of *in vitro* substrate specificity of S8H enzyme.

Table S6. Chemical analysis of soil mixes originating from different batches used in two experimental replicates.

Supplementary Tables and Figures
