# Supplementary material for: Scopoletin 8-hydroxylase: a novel enzyme involved in coumarin biosynthesis and iron-deficiency responses in Arabidopsis
Source: J Exp Bot. 2018 Jan 18;69(7):1735–48. doi: 10.1093/jxb/ery005 (PMC5888981; doi:10.1093/jxb/ery005)
Supplement: Supplementary Tables and Figures [file ery005_suppl_supplementary_tables_and_figures.pdf]

SUPPLEMENTARY DATA to

Siwinska et al.

Supplementary Figures

Fig. S1. Schematic representation of independent T-DNA mutant lines for the *At3g12900* gene.

Fig. S2. Lack of *S8H* transcript in the *s8h-1* T-DNA mutant line confirmed by qRT-PCR.

Fig. S3. Multiple-sequence alignment of amino acid sequences of F6′H1, F6′H2, S8H and ANS enzymes.

Fig. S4. Results of recombinant His-tagged S8H protein purification.

Fig. S5. The optimal pH and temperature for *in vitro* activity of S8H.

Fig. S6. Characterization of *Arabidopsis thaliana* S8H enzyme activity.

Fig. S7. Kinetic parameters V_{max} and K_m of S8H.

Fig. S8. Phenotypic appearance of Col-0 and *s8h* plants grown *in vitro* in liquid cultures with various levels of Fe.

Fig. S9. Chlorophyll content and chlorophyll a/b ratio of Col-0 and *s8h* plants grown *in vitro* in liquid cultures with various levels of Fe.

Fig. S10. Phenotyping and biochemical characterization of Col-0 and *s8h-1* plants grown in soil mixes with different Fe availability.

Fig. S11. Trace element content of Col-0 and *s8h* plant roots grown *in vitro* in Fe-depleted liquid culture.

Fig. S12. Phenotypic appearance of 2-week-old Col-0 and *s8h-1* plants grown on 0.25 MS and 0.5 MS media.

Fig. S13. Fresh weight of 3-week-old Col-0 and *s8h* plants grown *in vitro* on 0.25 MS and 0.5 MS media.

Fig. S14. Phenotypic appearance of Col-0 and *s8h* plants observed under UV light.

Fig. S15. Phenotypic characterization of Col-0 and *s8h* plants grown in 1× Heeg hydroponic solution (fully changed once per week) with 0–40 μM Fe^{2+} content.

Fig. S16. Phenotypic characterization of Col-0 and *s8h* plants grown in 10× Heeg hydroponic solution (fully changed once per week) with 0–40 μM Fe^{2+} content.

Fig. S17. Phenotypic characterization of Col-0 and *s8h* plants grown in 1× Heeg hydroponic solution (refilled with a fresh medium) with 0–40 μM Fe^{2+} content.

Fig. S18. Phenotypic characterization of Col-0 and *s8h* plants grown in 10× Heeg hydroponic solution (refilled with a fresh medium) with 0–40 μM Fe^{2+} content.

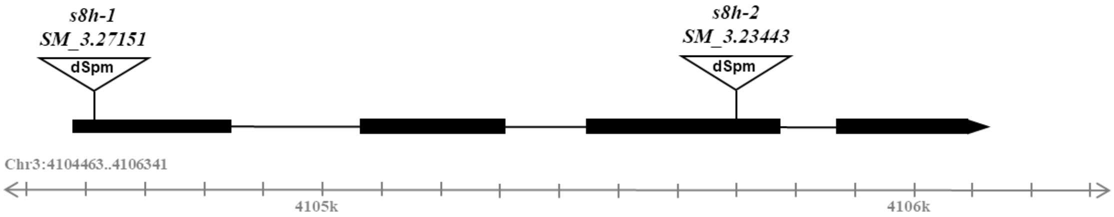

Fig. S1. Schematic representation of independent T-DNA mutant lines for the *At3g12900* gene. Gene structure of AtS8H with T-DNA insertion sites and map detail are indicated. Black boxes represent exons and solid lines represent introns of AtS8H.

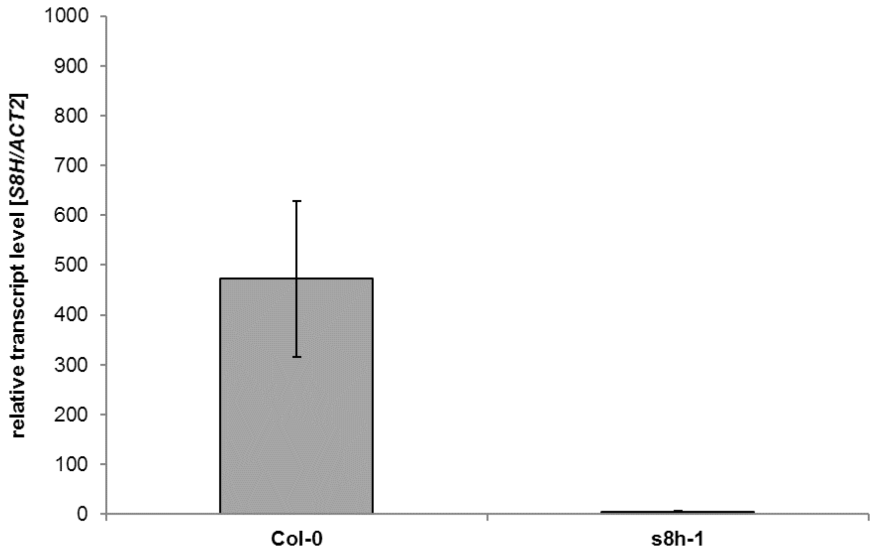

Fig. S2. Lack of *S8H* transcript in the *s8h-1* T-DNA mutant line confirmed by qRT-PCR. *S8H* gene expression level determined by qPCR was normalised to the reference gene *ACT2*. Col-0 plants were used as a positive control. Error bars represent the SD from three measurements.

```
F6'H1           MAPTLLTTQFSNPAEVTDFVVYKGNGVKGLSETG-IKALPEQYIQPLEERL-INKFVN-- 56
F6'H2           MNQT-LAAQFLTRDQVTNFVVHEGNGVKGLSETG-IKVLPDQYIQPFEERL-INFHVKE- 56
S8H             -----MGINFEDQTTLFNFVVREGNGVKGMIDSG-LSSVPRPFVQPLSERIPTQKALTC- 53
ANS             --------------------MVAVERVESLAKSG-IISIPKEYIRPKEELESINDVFLEE 39
                                    :   : *:.: .   :  :*  :::* .*    :  .

F6'H1           --ETDEAIPVIDMSNPD------EDRVAEAVCDAAEKWGFFQVINHGVPLEVLDDVKAAT 108
F6'H2           --DSDESIPVIDISNLD------EKSVSKAVCDAAEEWGFFQVINHGVSMEVLENMKTAT 108
S8H             --EATQPIDLSNLDGPQ------HKEVAKQIVEAAETLGFFQVVNHGVSVELLELLKSSA 105
ANS             KKEDGPQVPTIDLKNIESDDEKIRENCIEELKKASLDWGVMHLINHGIPADLMERVKKAG 99
                       :   ::.. :      .    . : .*:   *.::::***:.   :. :  :

F6'H1           HKFFNLPVEEKRKFTKENSLSTTVRFGTSFSPLAEQALEWKDYLSLFFVSE-AEAEQFWP 167
F6'H2           HRFFGLPVEEKRKFSREKSLSTNVRFGTSFSPHAEKALEWKDYLSLFFVSE-AEASQLWP 167
S8H             HEFFAQAPEEKSMYLKEVSPSKLVKYGTSFVPDKEKAIEWKDYVSMLYTND-SEALQHWP 164
ANS             EEFFSLSVEEKEKYANDQATGKIQGYGSKLANNASGQLEWEDYFFHLAYPEEKRDLSIWP 159
                . **  . **:  : .  : .      :.:    .  :**.*:.      :       **

F6'H1           DI---CRNETLEYINKSKKMVRRLLEYLGKNLNVKE--LDETKESLFMGSIRVNLNYYPI 222
F6'H2           DS---CRSETLEYMNETKPLVKKLLRFLGENLNVKE--LDKTKESFFMGSTRINLNYYPI 222
S8H             QP---CREVALEFLNSSMEMVKNVVNILMENVGVT---LEEEKMNGLMGTKMVNMNYYPT 218
ANS             KTPSDYIEATSEYAKCLRLLATKVFKALSVGLGLEPDRLEKEVGGLEELLLQMKINYYPK 219
                .      . . :. :    :. .:.. *  .:.     :::           :::****

F6'H1           CPNPDLTVGVGRHSDVSSLTILLQDQ--IGGLHVRSLASGN-WVHVPPVAGSFVINIGDA 279
F6'H2           CPNPELTVGVGRHSDVSSLTILLQDE--IGGLHVRSLTTGR-WVHVPPISGSLVINIGDA 279
S8H             CPSPELTVGVGRHSDMGMLTVLLQDG--IGGLYVK-LDNGE-WAEIPPVHGALVINIGDT 274
ANS             CPQPELALGVEAHTDVSALTFILHNM----VPGLQLFYEGK-WVTAKCVPDSIVMHIGDT 274
                **.*.*::*.  *:*:. **.:*::         :    *  *     : .::*::***.

F6'H1           MQIMSNGLYKSVEHRVLANGYNNRISVPIFVNP-KPESVIGPLPEVIA-NGEEPIYRDVL 337
F6'H2           MQIMSNGRYKSVEHRVLANGSYNRISVPIFVSP-KPESVIGPLLEVIE-NGEKPVYKDIL 337
S8H             LQILSNGKYKSAEHRVRTTNIGSRVSVPIFTAP-NPSQKVGPLPEVVK-RDGVARYKEFL 332
ANS             LEILSNGKYKSILHRGLVNKEKVRISWAVFCEPPKDKIVLKPLPEMVSVESPAKFPPRTF 334
                ::*:**. *:*  *   ..    *:* .:*  *   .  : ** *::  ..        .

F6'H1           YSDYVKYFFRKAHDGKKTVDYAKI- 361
F6'H2           YTDYVKHFFRKAHDGKKTIDFANI- 361
S8H             FQDYMNNFFGQPHDGKKSLDFARAE 357
ANS             AQHIEHKLFGKEQEELVSEKND--- 356
                  .  : :* : ::   : .
```

Fig. S3. Multiple-sequence alignment of amino acid sequences of F6′H1, F6′H2, S8H and ANS enzymes. The alignment was performed using the CLUSTALW (Thompson et al., 1994). All amino acid sequences, including F6′H1 and F6′H2 involved in scopoletin biosynthesis, S8H involved in fraxetin biosynthesis and ANS (anthocyanidin synthase of known crystal structure) involved in anthocyanin biosynthesis, were downloaded from TAIR (http://arabidopsis.org/). Residues that are conserved in F6′H1, F6′H2 and S8H sequences are highlighted in grey. Residues coloured in red correspond to amino acids biding Fe^{2+} (His 235, His 293, Asp 237 in F6′H1 sequence) and residues in green correspond to amino acids biding 2OG (Arg 303 and Ser 305 in F6′H1 sequence), which are highly conserved in all four analysed sequences. While blue colour in ANS sequence indicate amino acids biding *trans*-dihydroquercetin (DHQ) (Tyr 217, Tyr 142, Phe 304, Glu 306, Phe 144, Ile 122, Val 235, Phe 334, Ile 338, Leu 342, Thr 233, Lys341).

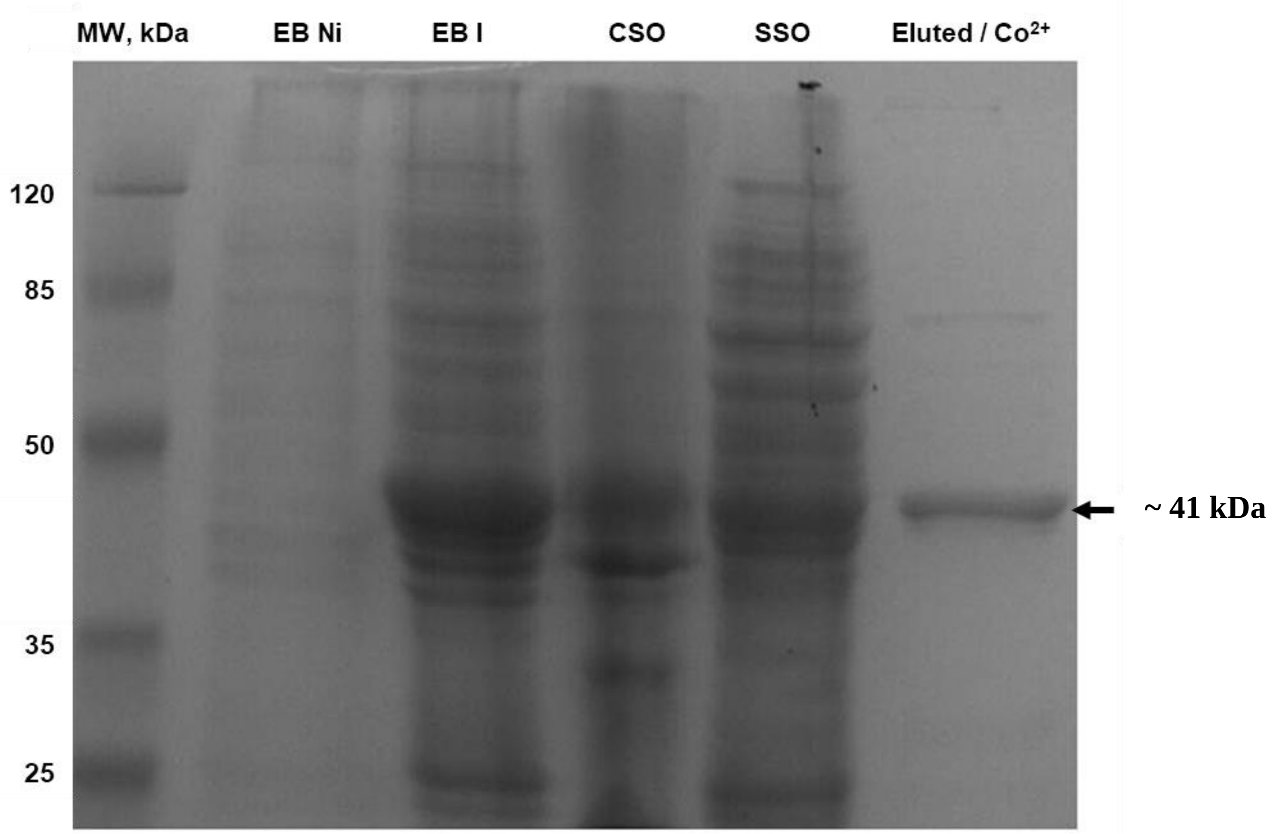

Fig. S4. Results of recombinant His-tagged S8H protein purification. The purification of soluble recombinant his-tagged proteins was done using the TALON Metal Affinity Resin (TAKARA) as described by the supplier. The purified proteins were eluted with 0.1 M potassium phosphate buffer, 200 mM imidazole solution (pH 8.0). The predicted molecular weight of the his-tagged S8H protein is ~ 41 kDa. (MW) molecular weight marker; (EBNi) crude extracts, non-induced cultures; (EBi) crude extracts, induced cultures; (CSO) sonification pellet, (SSO) sonification supernatant, (Eluted Co^{2+}) purified protein.

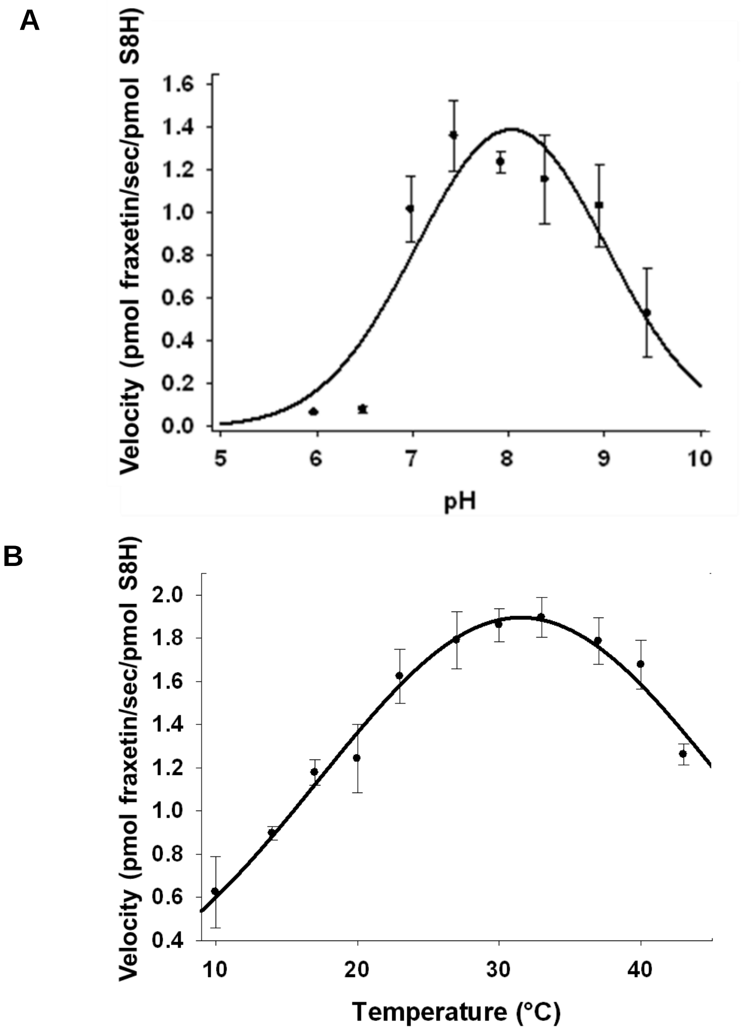

Fig. S5. The optimal pH and temperature for *in vitro* activity of S8H. (A) pH dependent activity of *Arabidopsis thaliana* S8H. The reactions were performed in triplicate at optimal temperature of 31.5 °C at 200 µM of scopoletin, 5 mM of alpha-acetoglutarate and 0.2 mM $FeSO_4$. pH range was 5.02-9.45 in buffer Tris 0.1 M. Reactions were monitored at 340 nm and scopoletin product was quantified with a standard curve. Nonlinear regression analysis against equation $f = a*exp(-0.5*[(x-x_0)/b)^2]$ was performed with Sigma Plot 12.0 Software. Optimal pH was 8.0 +/- 0.1. **(B)** Temperature dependent activity of Arabidopsis S8H. The reactions were performed in triplicate at optimal pH of 8.0 at 200 µM of scopoletin, 5 mM of alpha-acetoglutarate and 0.2 mM $FeSO_4$. Temperature range was 10-43 °C in buffer Tris 0.1 M. Reactions were monitored at 338 nm and fraxetin product was quantified with a standard curve. Nonlinear regression analysis against equation $f = a*exp(-0.5*[(x-x_0)/b)^2]$ was performed with Sigma Plot 12.0 Software. Optimal temperature was 31.5 +/- 0.4 °C.

A

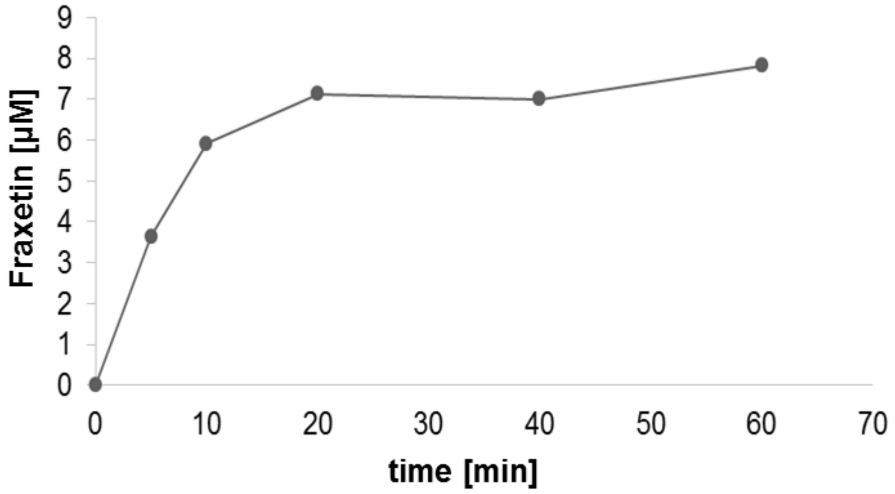

B

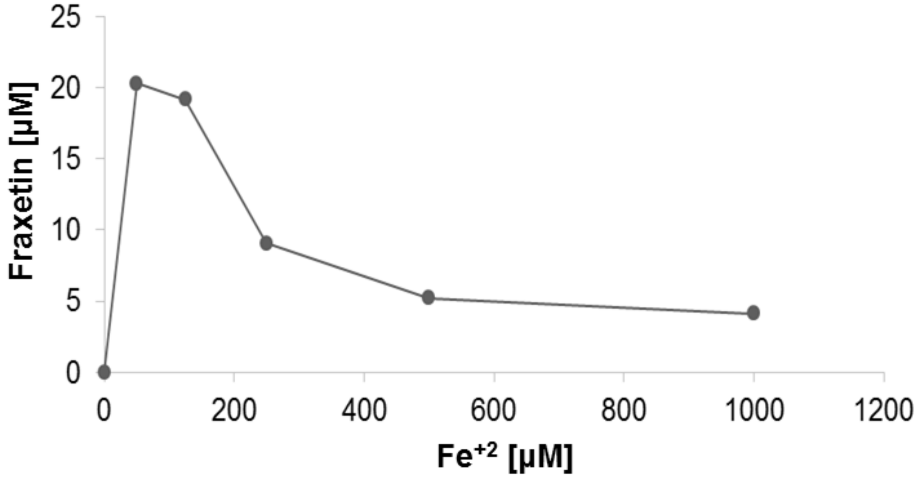

Fig. S6. Characterization of *Arabidopsis thaliana* S8H enzyme activity. (A) Correlation between incubation time of scopoletin with S8H enzyme and fraxetin biosynthesis. The optimal incubation time of the reaction mixture was set at 10 minutes. **(B)** Correlation between Fe^{2+} ion concentration (in the concentration range 0-1000 µM), S8H enzyme activity and the biosynthesis of fraxetin quantified with HPLC. The optimal concentration of ferrous ions in the reaction mixture was equal to 50 µM. Graphs were done using MS Excel software.

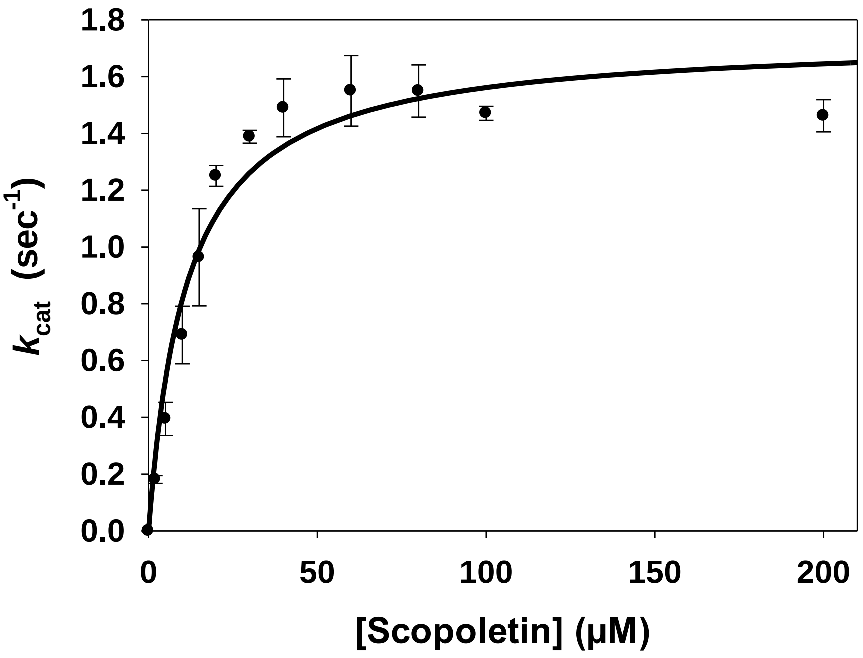

Fig. S7. Kinetic parameters *V_{max}* and *K_m* of S8H. The reactions were performed in triplicate at optimal pH of 8.0. Scopoletin concentration was in the range of 2 - 200 µM with 5 mM of alpha-acetoglutarate and 0.2 mM $FeSO_4$. Temperature was set at 31.5 °C in buffer Tris 0.1 M. Reactions were monitored at 340 nm and fraxetin product was quantified with a standard curve. Regression analysis was performed against Michaelis Menten relation with Sigma Plot 12.0 Software. Catalytic parameters were Vmax = 1.73 +/- 0.09 pmol fraxetin/sec/pmol S8H and *Km* = 11 +/- 2 µM.

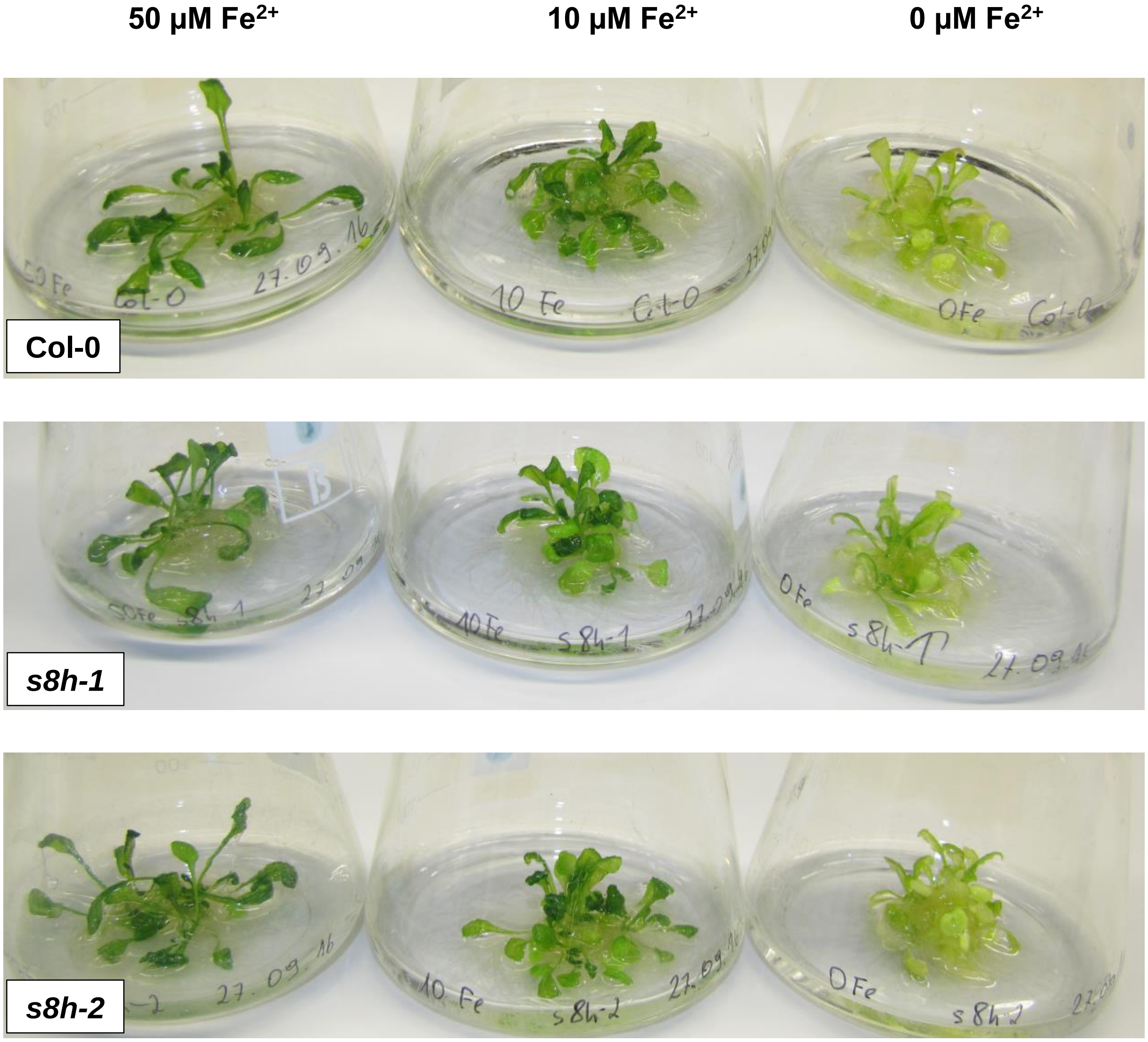

Fig. S8. Phenotypic appearance of Col-0 and *s8h* plants grown *in vitro* in liquid cultures with various levels of Fe. 10-days-old seedlings grown on 0.25 MS medium were transferred from agar plates to the 250 ml Erlenmeyer flasks. Flasks containing 0.25 MS with various Fe availability (0, 10 and 50 µM Fe^{2+}) were kept on rotary platform shakers at 120 rpm for next three weeks until the optimal growth of roots was observed. The plant material was harvested, growth media were collected and subsequently used for metabolic profiling and trace elements analysis. Plants were grown in growth chamber under a photoperiod of 16 h light (~5000 lux) at 22 °C and 8 h dark at 20 °C.

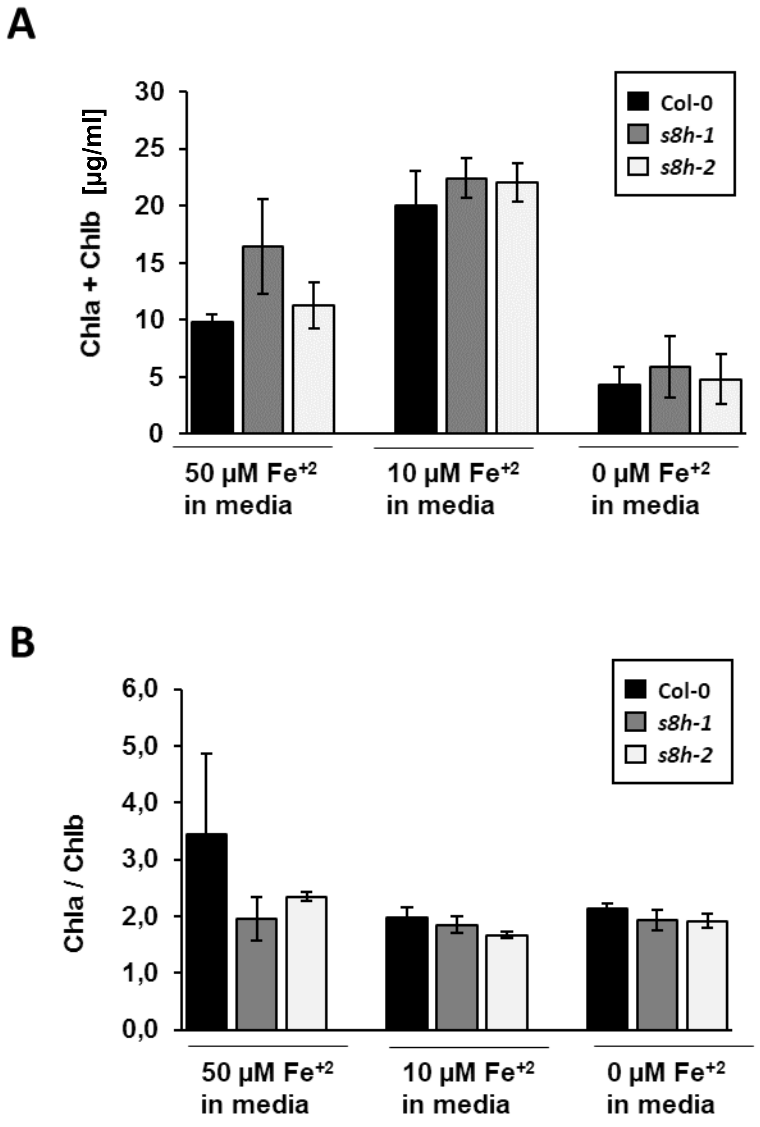

Fig. S9. Chlorophyll content and chlorophyll a/b ratio of Col-0 and *s8h* plants grown *in vitro* in liquid cultures with various levels of Fe. Growth conditions are described in detail in Fig. S8.

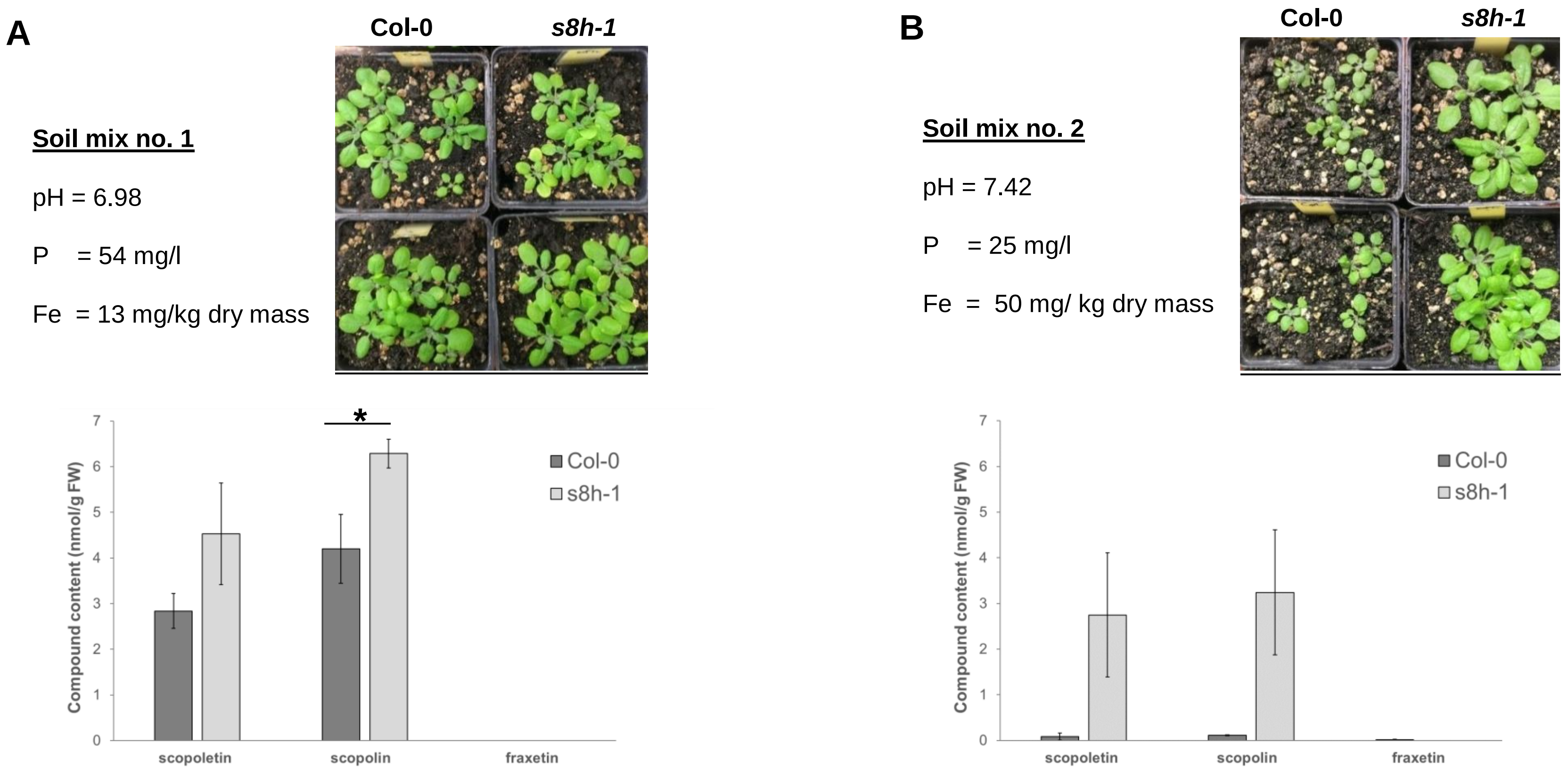

Fig. S10. Phenotyping and biochemical characterization of Col-0 and *s8h-1* plants grown in soil mixes with different Fe availability. Plant phenotypes and the corresponding metabolic profiles (scopoletin, scopolin, and fraxetin levels quantified by UHPLC) of root extracts of plants grown **(A)** in soil mix no. 1 and **(B)** soli mix no. 2 (Table S6). The latter soil is characterized by a higher pH, lower phosphorus (P) level and higher Fe content when compared with soil mix no. 1. The rosettes of *s8h* mutants were visible larger compared to Col-0 plants once grown in a soil mix no. 2. The UHPLC analysis indicated that *s8h-1* mutants accumulated higher levels of scopoletin and its corresponding glycoside scopolin than Col-0 plants. These differences were particularly striking in a slightly alkaline soil (soil mix no. 2). Error bars represent the SD from three measurements. (*) $p < 0.05$. (FW) fresh weight.

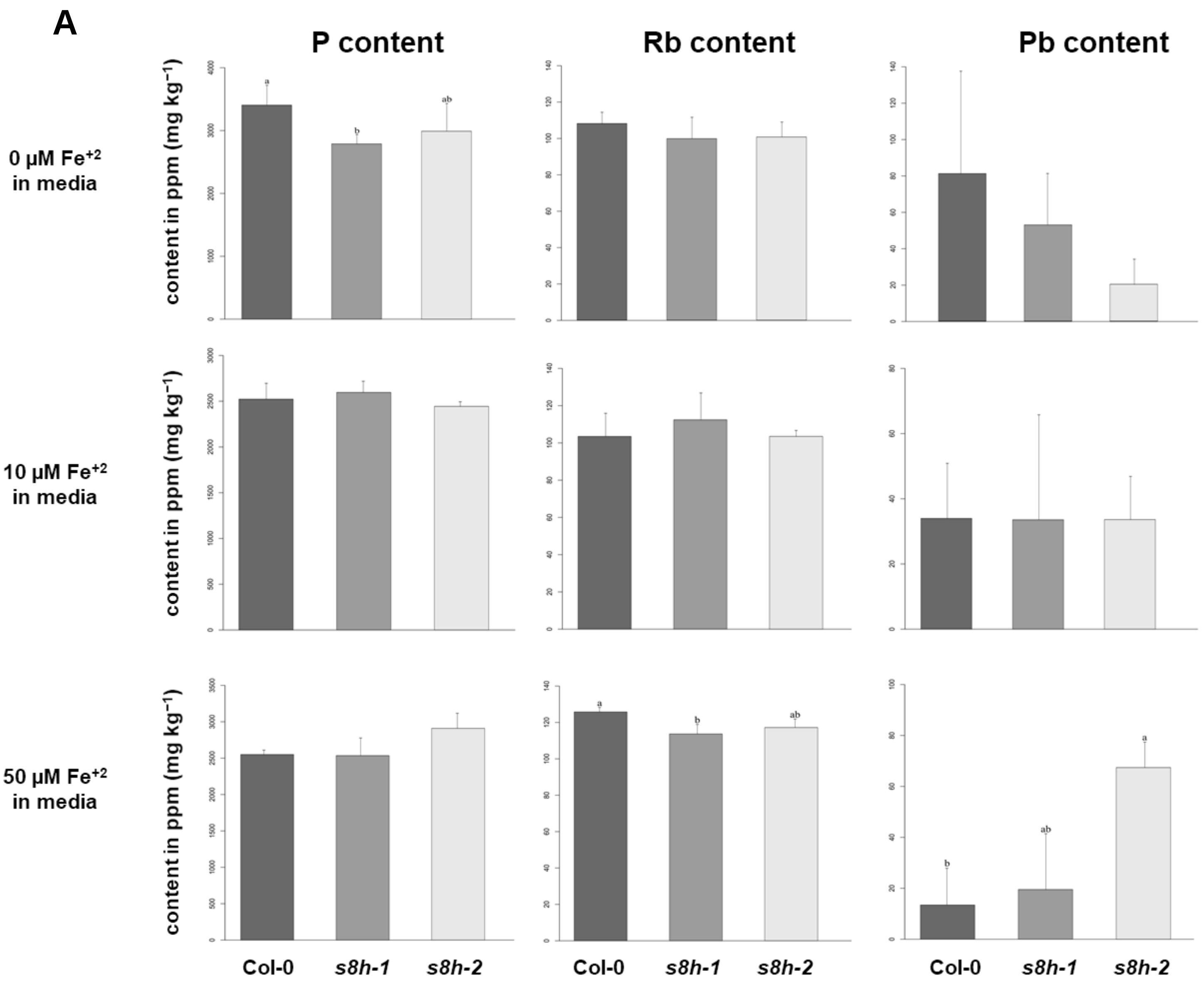
A
P content
Rb content
Pb content
0 µM Fe+2
in media
10 µM Fe+2
in media
50 µM Fe+2
in media
content in ppm (mg kg-1)
Col-0
s8h-1
s8h-2

B

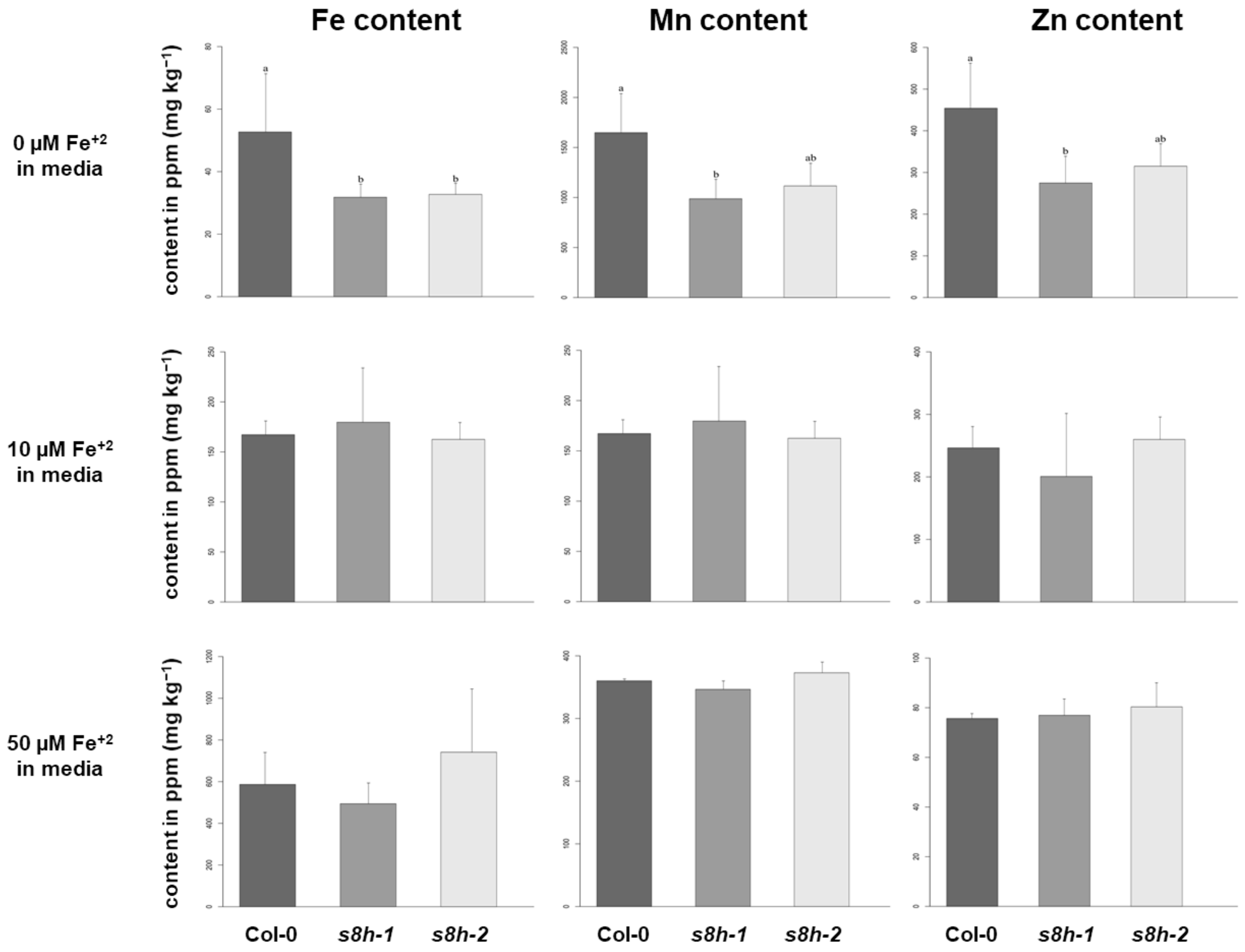
Fe content
Mn content
Zn content
0 µM Fe+2 in media
10 µM Fe+2 in media
50 µM Fe+2 in media
content in ppm (mg kg−1)
Col-0
s8h-1
s8h-2

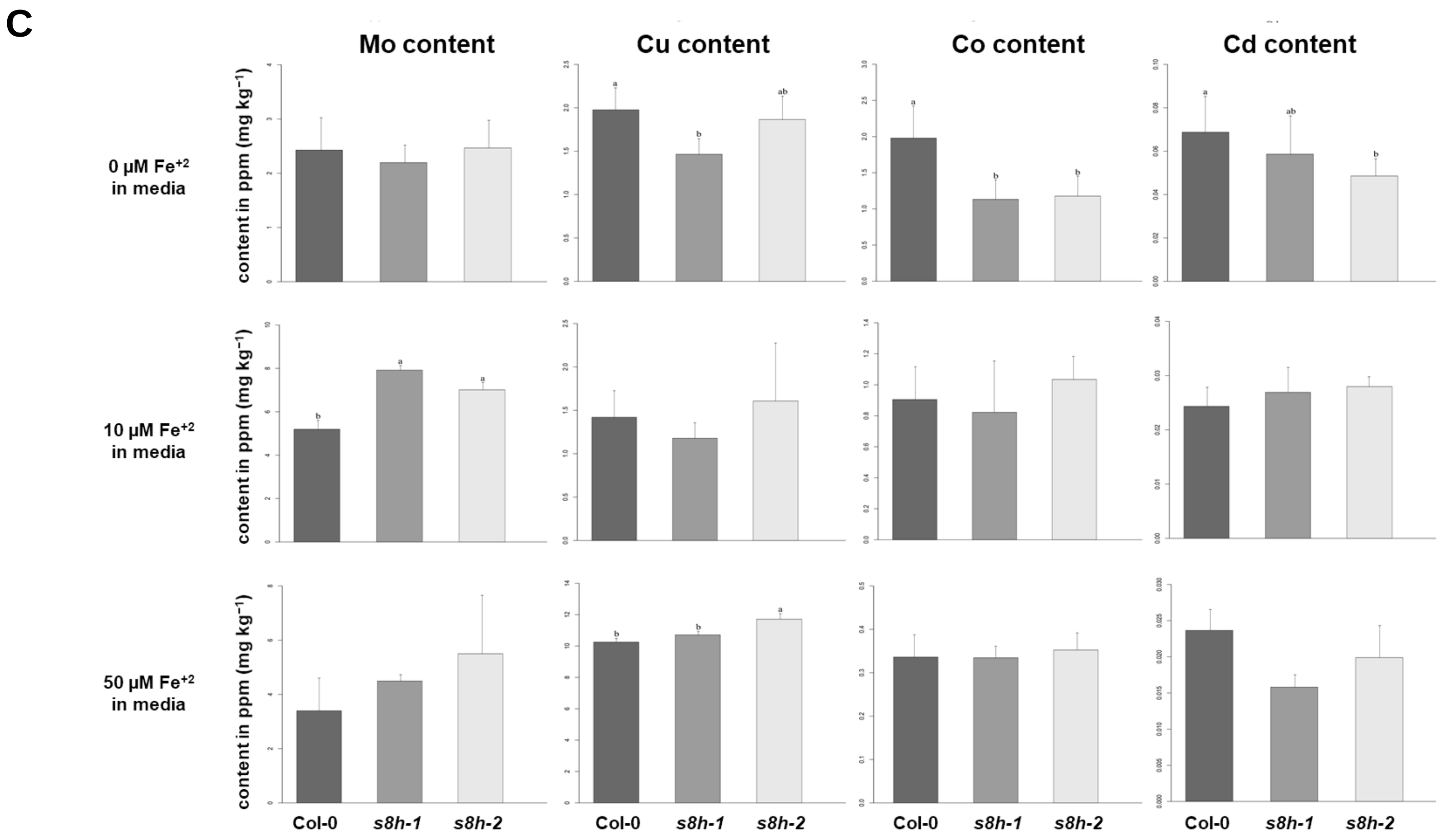

Fig. S11. Trace element content of Col-0 and *s8h* plant roots grown *in vitro* in Fe-depleted liquid culture. Concentrations of trace elements shown in ppm (mg kg^{-1}) were determined by Inductively Coupled Plasma-Mass Spectrometry (ICP-MS). The results of one representative experimental replicate are presented. Error bars represent the SD from five biological replicates for plants grown at 0 µM Fe^{2+} and three biological replicates for plants grown at 10 and 50 µM Fe^{2+}. Values that are significantly different are indicated by different letters. In all tests p-value < 0.05 was used.

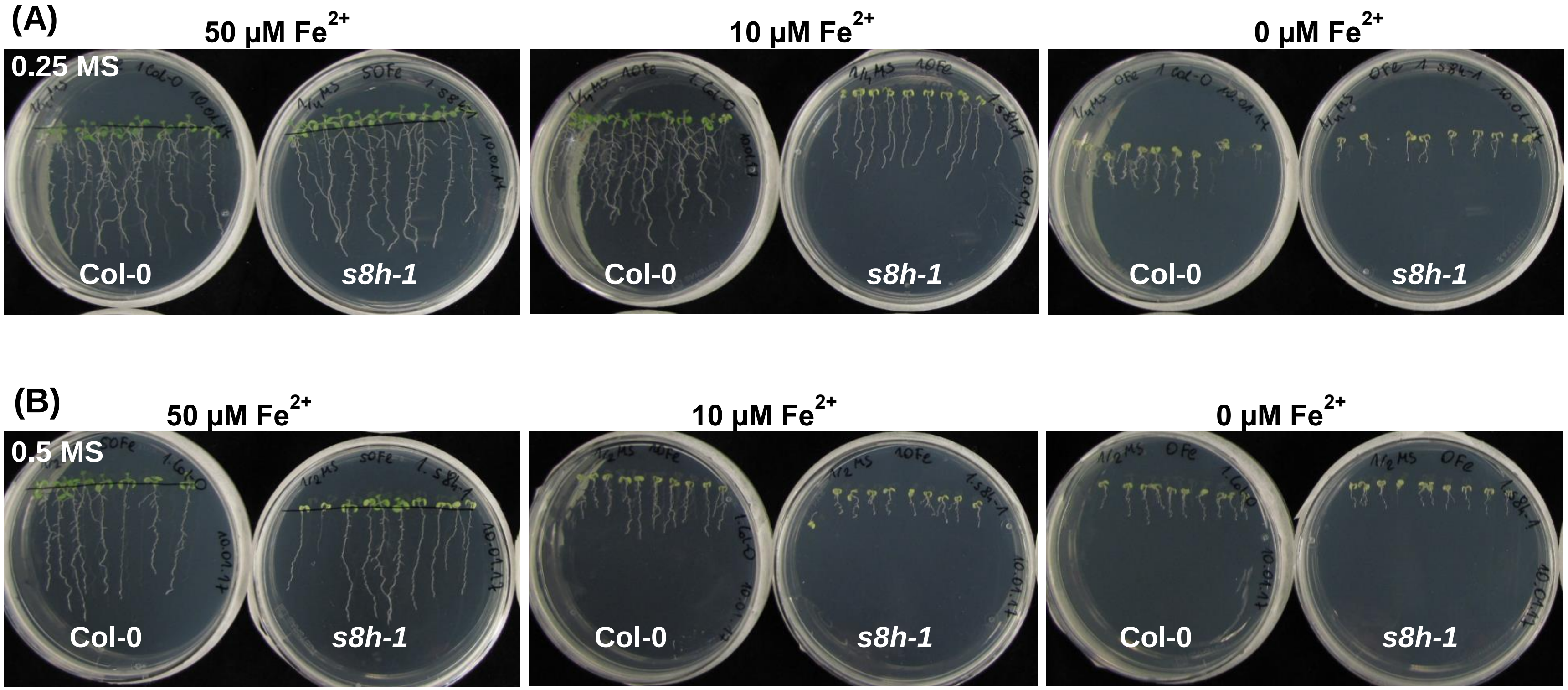

Fig. S12. Phenotypic appearance of 2-week-old Col-0 and *s8h-1* plants grown on 0.25 MS and 0.5 MS media. Plants were grown Murashige and Skoog's (MS) media with various Fe availability (0-50 µM Fe^{2+}) in plant growth chambers under a photoperiod of 16 h light (~5000 lux) at 22 °C and 8 h dark at 20 °C. Due to the limited space, only *s8h-1* line is shown, a second mutant allele (*s8h-2*) showed a very similar respond.

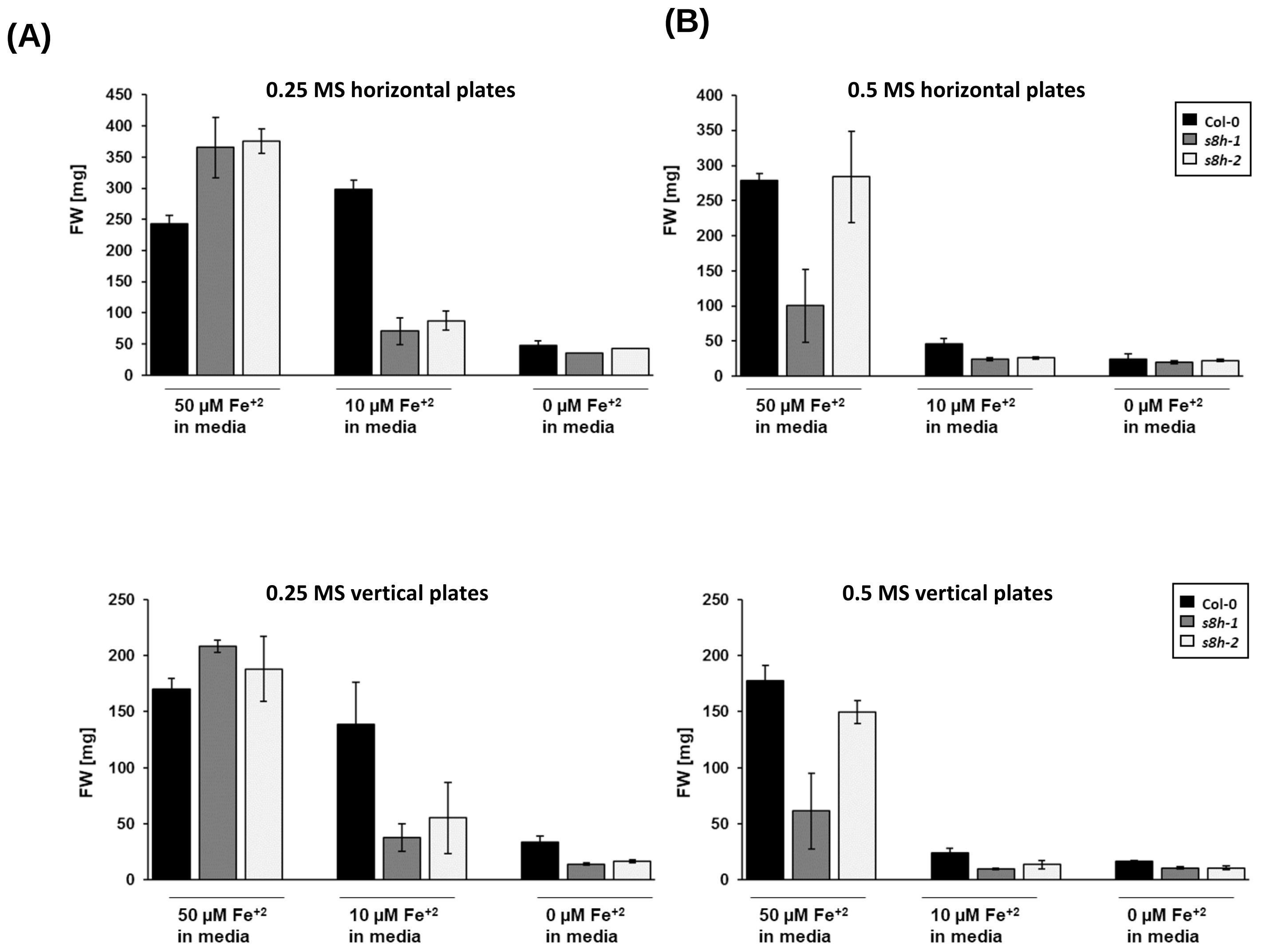

Fig. S13. Fresh weight of 3-week-old Col-0 and *s8h* plants grown *in vitro* on 0.25 MS and 0.5 MS media. Plants were grown on plates with various Fe availability (0-50 µM Fe^{2+}) placed in either a vertical or horizontal position, in plant growth chambers under a photoperiod of 16 h light (~5000 lux) at 22 °C and 8 h dark at 20 °C.

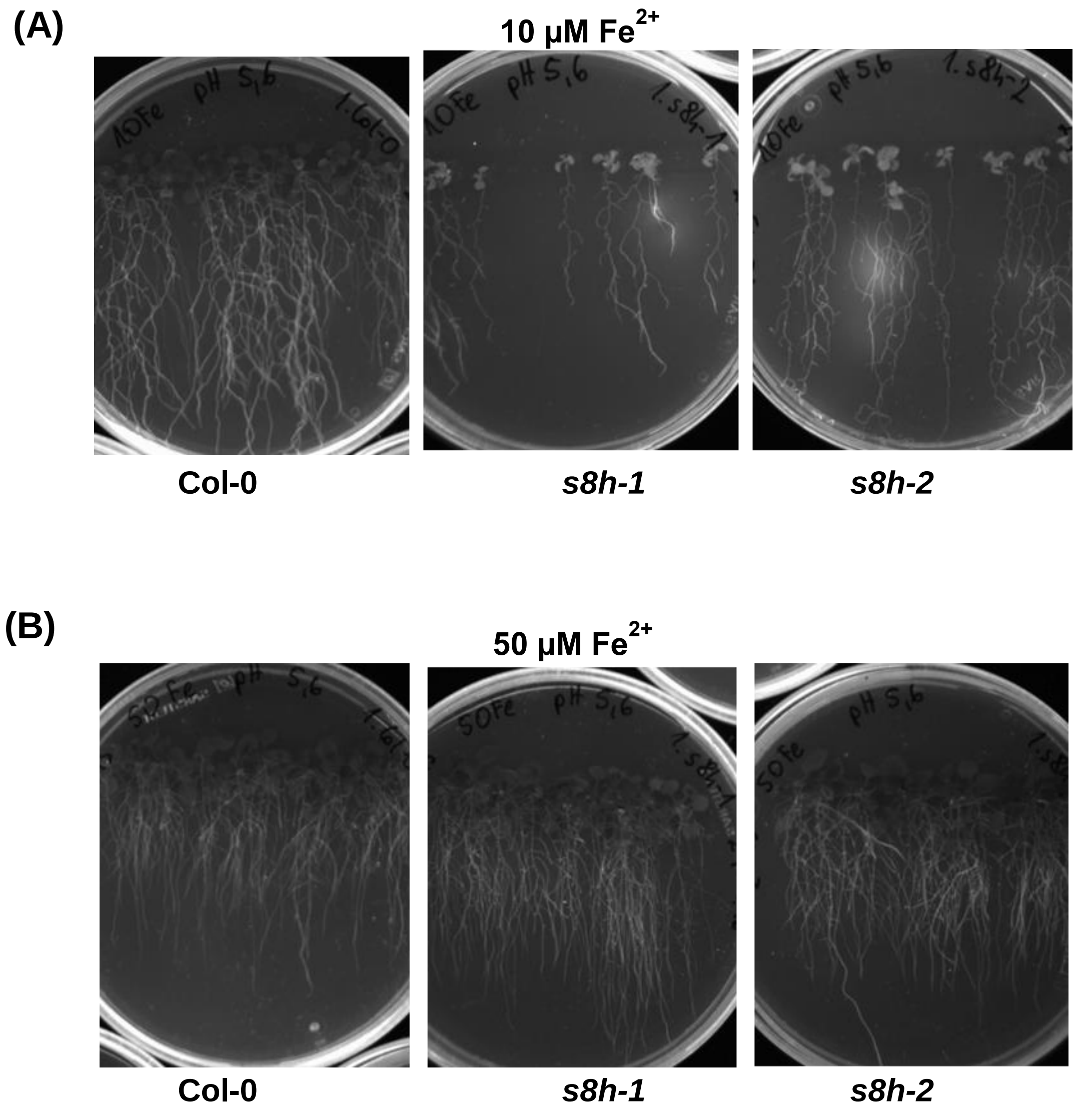

Fig. S14. Phenotypic appearance of Col-0 and *s8h* plants observed under UV light. Plants were grown on MS media containing **(A)** 10 μM Fe^{2+} or **(B)** 50 μM Fe^{2+}. Under Fe-deficient conditions (A), mutant plants secreted an increased level of fluorescent compound.

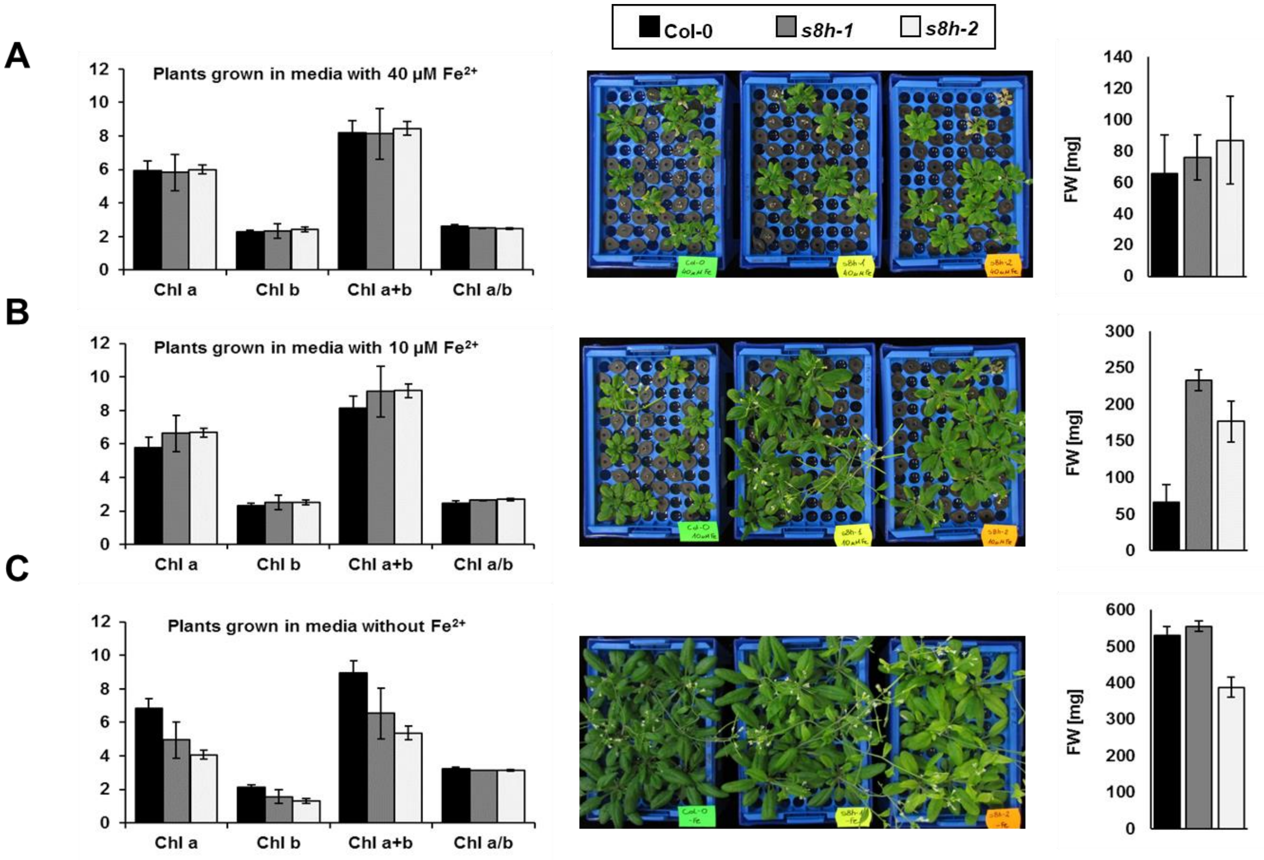

Fig. S15. Phenotypic characterization of Col-0 and *s8h* plants grown in 1× Heeg hydroponic solution (fully changed once per week) with 0–40 µM Fe^{2+} content. After three weeks of growth in optimal hydroponic solution (40 µM Fe^{2+}) plants were transferred to solutions with various Fe availability (A-C) and cultured until chlorosis became visible. Hydroponic solutions were fully changed once per week. Leaf chlorophyll content (µg/ml) and fresh weight (mg) were calculated. The results of one representative experimental replicate are presented. Error bars represent the SD from three biological replicates for chlorophylls and six to nine biological replicates for fresh weight (FW).

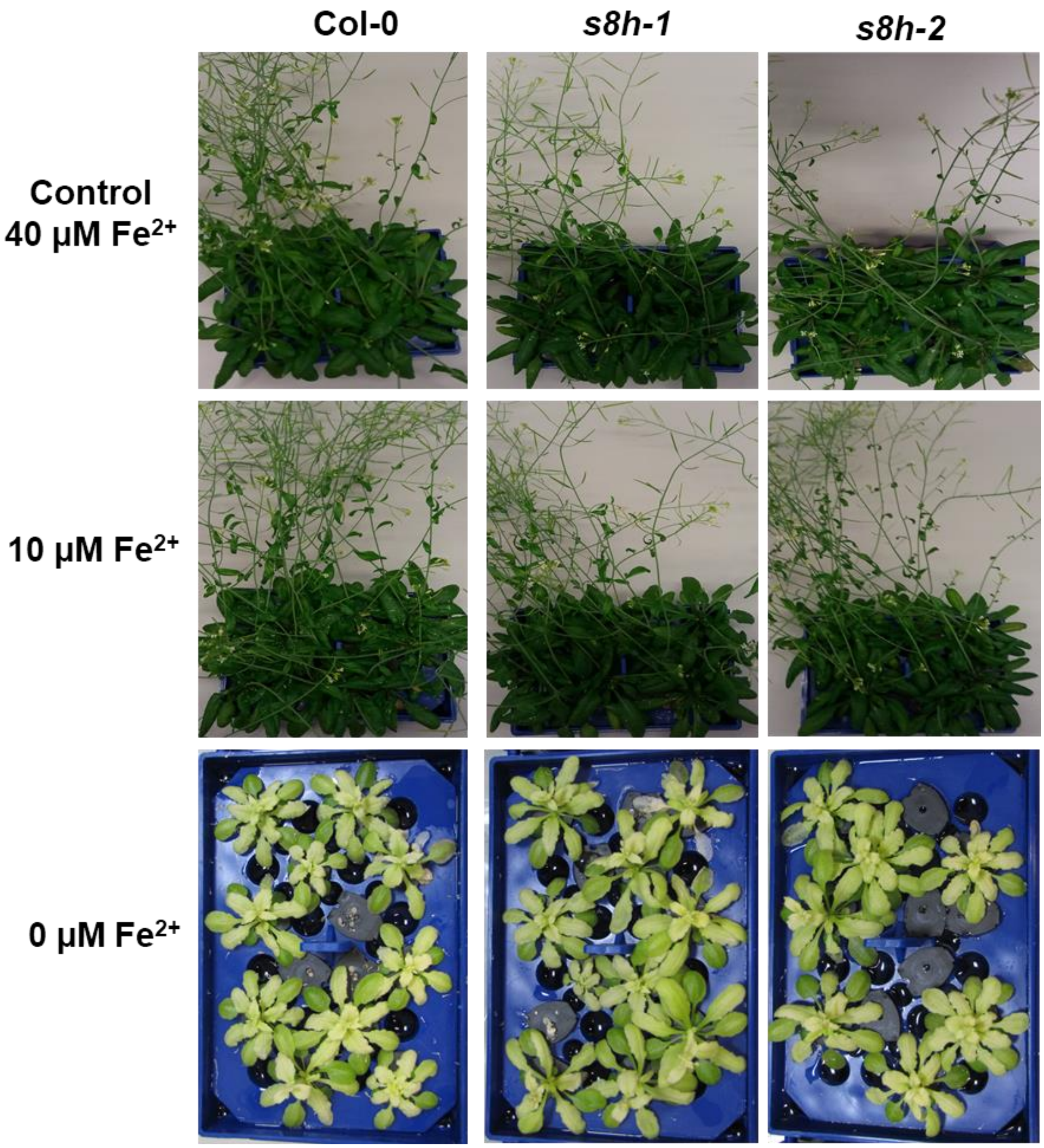

Fig. S16. Phenotypic characterization of Col-0 and *s8h* plants grown in 10× Heeg hydroponic solution (fully changed once per week) with 0–40 µM Fe^{2+} content. After three weeks of growth in optimal hydroponic solution (40 µM Fe^{2+}) plants were transferred to solutions with various Fe availability and cultured until chlorosis became visible. Hydroponic solutions were fully changed once per week. The results of one experimental replicate are presented.

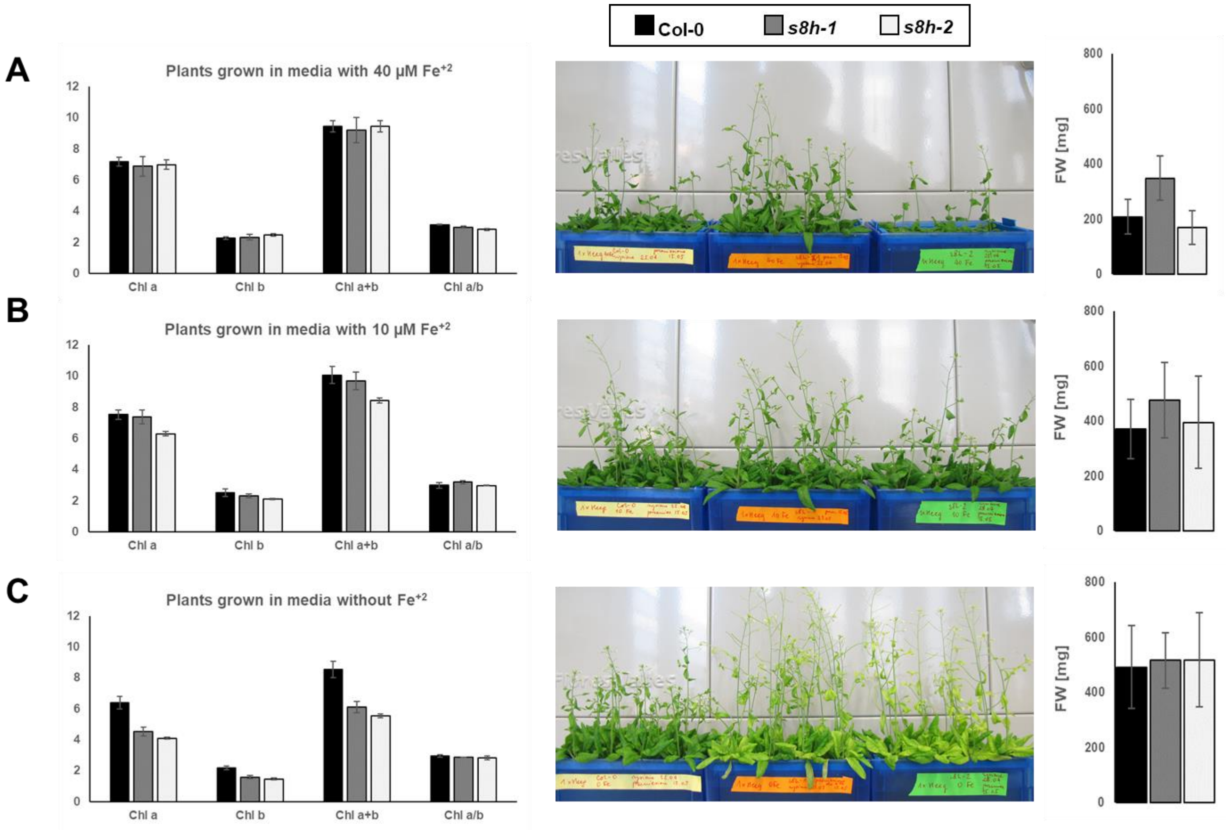

Fig. S17. Phenotypic characterization of Col-0 and *s8h* plants grown in 1× Heeg hydroponic solution (refilled with a fresh medium) with 0–40 µM Fe^{2+} content. After three weeks of growth in optimal hydroponic solution (40 µM Fe^{2+}) plants were transferred to solutions with various Fe availability (A-C) and cultured until chlorosis became visible. Boxes were refilled by the addition of a fresh medium to keep the similar volume of solution in each culture. Leaf chlorophyll content (µg/ml) and fresh weight (mg) were calculated. The results of one representative experimental replicate are presented. Error bars represent the SD from three biological replicates (chlorophylls) or 14-21 replicates (FW).

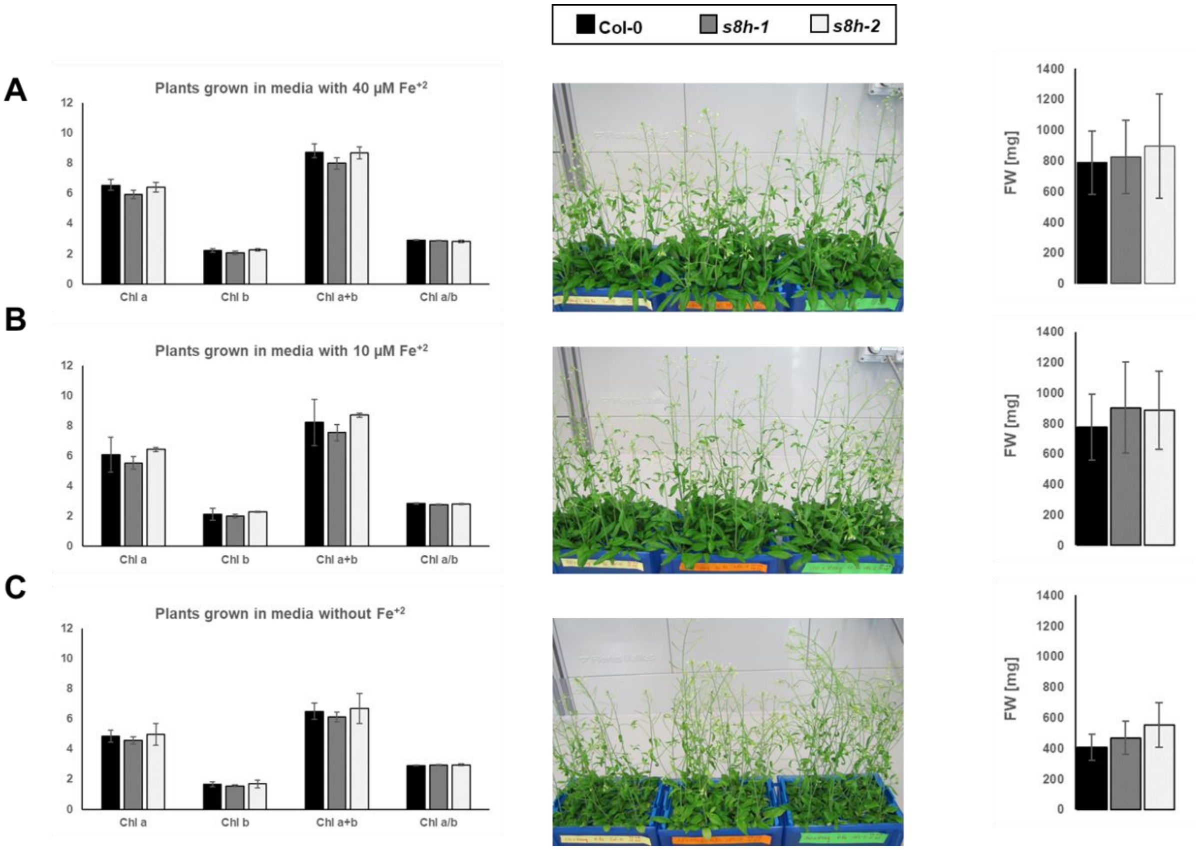

Fig. S18. Phenotypic characterization of Col-0 and *s8h* plants grown in 10× Heeg hydroponic solution (refilled with a fresh medium) with 0–40 µM Fe^{2+} content. After three weeks of growth in optimal hydroponic solution (40 µM Fe^{2+}) plants were transferred to solutions with various Fe availability (A-C) and cultured until chlorosis became visible. Boxes were refilled by the addition of a fresh medium to keep the similar volume of solution in each culture. Leaf chlorophyll content (µg/ml) and fresh weight (mg) were calculated. The results of one representative experimental replicate are presented. Error bars represent the SD from three biological replicates (chlorophylls) or 14-18 replicates (FW).

Supplementary Tables

Table S1. Genotyping of *s8h* mutant lines.

Table S2. Lack of *S8H* transcript in the *s8h* mutant backgrounds.

Table S3. Modified Heeg solutions used in hydroponic cultures.

Table S4. PCR amplification of the *S8H* ORF.

Table S5. Compounds used in the analysis of *in vitro* substrate specificity of S8H enzyme.

Table S6. Chemical analysis of soil mixes originating from different batches used in two experimental replicates.

Table S1. Genotyping of *s8h* mutant lines. Genotyping of *s8h-1* T-DNA line (SM_3.27151) was done using PCR amplification with Dream*Taq* Green PCR Master Mix (2X) (Thermo Scientific) according to the manufacturer protocol and primers described below. Amplified fragments were sequenced using the same primers and BigDye® Terminator v3.1 (Life Technologies). Sequencing reaction products were separated and analyzed by 3730xl DNA Analyzer (Genomed). All sequences were analysed by BLAST (http://blast.ncbi.nlm.nih.gov/Blast.cgi). A second allelic T-DNA mutant line of S8H (SM_3.23443), *sh8-2*, was genotyped using PCR amplification with GXL DNA Polymerase (Takara) according to the manufacturer protocol and the primers presented below. Primers were designed using the software available on the website http://signal.salk.edu/tdnaprimers.2.htm.

**Gene ID (allele) (T-DNA line)**	**Primer name**	**Primer sequence**
AT3G12900 (*s8h-1*) (SM_3.27151)	SM_3.27151_left	5'-GCGAGACGCATGCACCACAA-3'
	SM_3.27151_right	5'-TGTTCACGCTCATGTATAGCTGGT-3'
	SM_LB	5’- TACGAATAAGAGCGTCCATTTTAGAGTGA-3’
AT3G12900 (*s8h-2*) (SM_3.23443)	SM_3.23443_1	5'- TGTGCCAGATAAGGAGAAGGCGA -3'
	SM_3.23443_2	5'- CGGCACGTGCGAAGTCGAGA -3'
	SM_LB	5’- TACGAATAAGAGCGTCCATTTTAGAGTGA-3’

Table S2. Lack of *S8H* transcript in the *s8h* mutant backgrounds. Selected *s8h-1* homozygous mutant lines were tested for *S8H* gene expression by a quantitative real-time PCR analysis. Total RNA was extracted from T-DNA mutants and Col-0 lines as described in PCR amplification of *S8H* ORF in Table S4. 1 µg of RNA was used for reverse transcription by Maxima First Strand cDNA Synthesis Kit (Thermo Scientific) with oligo(dT)18 primers and random hexamer primers. qRT-PCR was performed using LightCycler® 480 Real-Time PCR System (Roche) and Luminaris HiGreen qPCR Master Mix (Thermo Scientific) on 96-well plates (4titude®), using the gene-specific primers for *ACT2* (*ACTIN2*) (Czechowski et al., 2005) and *S8H* shown below. Primers specificity for *S8H* were confirmed by the analysis of the melting curves. Primers efficiency was analysed by standard curve and serial dilutions of cDNA used for the reaction. Relative transcript level (RLT) of the *S8H* was normalized to the transcript level of the house-keeping *ACTIN2* gene (At3g18780) and calculated as follows: RLT = N0 * 2Cp; N0 – initial copy number, Cp – crossing point. A lack of *S8H* transcript in the *s8h-2* mutant background was confirmed by RT-PCR using primers shown in Table S2.

**Method**	**Primer name**	**Primer sequence**
qPCR	*ACT2* (*ACTIN2*)	5’-CTTGCACCAAGCAGCATGAA-3’<br>3’-CCGATC CAGACACTGTACTTCCTT-5’
qPCR	*S8H*	5’-GCCGAGACACTTGGCTTCTT-3’<br>5’-CAGCAGCTCCACCGAAACA-3’
RT-PCR	*ACT2* (*ACTIN2*)	5’-TCCCAGTGTTGTTGGTAGGC-3’<br>5’-CAAGACGGAGGATGGCATGA-3’
RT-PCR	SM_3.23443_1<br>SM_3.23443_3	5'- TGTGCCAGATAAGGAGAAGGCGA-3’<br>5’-GAACCCTATGCTCCGCACTT-3’
RT-PCR	SM_3.23443_1<br>SM_3.23443_4	5’-TGTGCCAGATAAGGAGAAGGCGA-3’<br>5’-TTCTGGGAGGGATTTGGTGC-3’

Table S3. Modified Heeg solutions used in hydroponic cultures.

	10 x Heeg	1 x Heeg
**Macronutrients**	**Final conc. (mM)**	**Final conc. (mM)**
KNO_3	2	2
$Ca(NO_3)_2$•$4H_20$	2	2
$MgSO_4$	0.5	0.5
NH_4NO_3	0.4	0.4
KH_2PO_4	0.25	0.25
**Micronutrients**	**Final conc. (uM)**	**Final conc. (uM)**
FeEDTA	40	40
H_3BO_3	25	2.5
$MnCl_2$•$4H_20$	2	0.2
$ZnSO_4$•$7H_20$	2	0.2
$CuSO_4$•$5H_20$	0.5	0.05
KCl	4.5	0.45
$(NH_4)_6Mo_7O_{24}$	0.08	0.008
$CoCl_2$	0.26	0.026

Table S4. PCR amplification of the *S8H* ORF. RNeasy® Plant Mini Kit (Qiagen) was used following the instructions of the manufacturer. 0.5 μg of RNA was used for reverse transcription by Maxima First Strand cDNA Synthesis Kit (Thermo Scientific).

**PCR Reaction Mixture (50 μl)**	**PCR Condition (35 cycles)**
cDNA (from roots RNA)	denaturation 98 °C / 3 min
2.5 U of LA Taq™ DNA Polymerase (Takara)	98 °C / 10 sec
200 μM dNTP	52 °C / 15 sec
1 μM primers	72 °C / 60 sec
1 × PCR Buffer containing 5 mM Mg^{2+}	extension 72 °C / 5 min

Table S5. Compounds used in the analysis of *in vitro* substrate specificity of S8H enzyme. (+) enzyme activity was observed for the selected compound, (-) lack of enzyme activity.

Compound	Activity	Structure	Compound	Activity	Structure
**feruloyl-CoA**	**-**	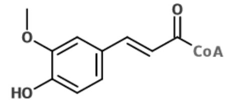	**6,7-dimethoxycoumarin (scoparon)**	**-**	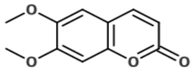
**coumaryl-CoA**	**-**	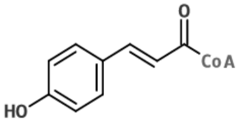	**isoscopoletin**	**-**	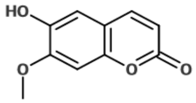
**cinnamyl-CoA**	**-**	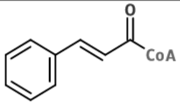	**6-methoxycoumarin**	**-**	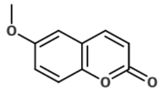
**caffeoyl-CoA**	**-**	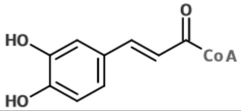	**7-methoxycoumarin**	**-**	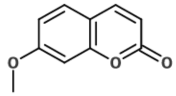
**limetin**	**-**	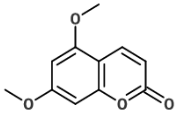	**7,8-dihydroxycoumarin (daphnetin)**	**-**	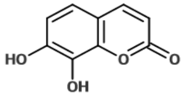
**esculetin**	**-**	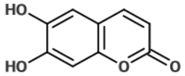	**esculin**	**-**	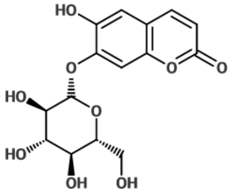

**fraxetin**	-	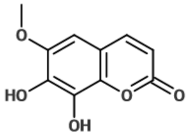	**fraxin**	-	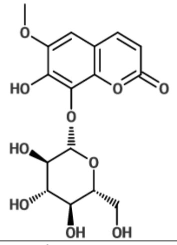
**scopoletin**	+	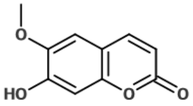	**scopolin**	-	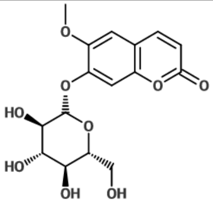
**umbelliferone**	-	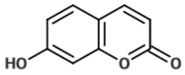	**skimmine**	-	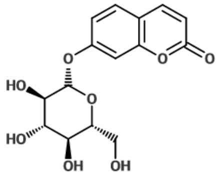
**coumarin**	-	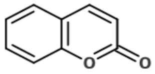			

Table S6. Chemical analysis of soil mixes originating from different batches used in two experimental replicates. Arabidopsis seeds were stratified on water-saturated Whatman paper in Petri dishes for four days at 4 °C and subsequently were sown out in soil mixes previously described by Ihnatowicz et al. (2014). Plant were grown in controlled environment during a 16 h light (~9000 lumens per square meter (lux)) at 22 °C and 8 h dark at 20 °C.

In contrast to the **Soil Experiment 1**, in which *s8h* mutants were visible larger than Col-0 plants when grown in a soil mix no. 2 (Figure S9), no apparent phenotypic differences between *s8h-1* and WT plants were observed in a soil mix no. 2 used in the second experimental replicate (**Soil Experiment 2**). As the soil mixes used in both independent experiments originated from the same producer but different batches, we decided to elucidate their contents of macro- and micronutrients by conducting a chemical analysis (Regional AgroChemical Station in Gdansk, Poland (OSCh-R, http://www.oschrgdansk.pl/)). The soil mix no. 2 used in the first experiment (**Soil Experiment 1**) was characterized by a lower P level and a higher Fe content when compared with soil mix no. 2 used in the second experiment (**Soil Experiment 2**).

**Experimental replicate**		**Soil Experiment 1**		**Soil Experiment 2**
		**soil mix no. 1**	**soil mix no. 2**	**soil mix no. 2**
	**pH in H_2O**	6.98±0.35	7.42±0.37	7.56±0.38
	**Salinity [gNaCl/dm3]**	0.30±0.04	1.90±0.28	1.19±0.18
**Macronutrients [mg/dm3]**	**NO^3**	4.5±0.9	141±28	101±20
	**Cl**	<10.0*	29.6±7.4	25.2±6.3
	**P**	**54±8.1**	**25.0±3.8**	**56.0±8.4**
	**K**	110±16	79.4±11.9	77.0±11.6
	**Ca**	992±149	2731±410	3326±499
	**Mg**	362±54	373±56	472**
**Micronutrients [(mg/kg dry mass (d.m.)]**	**B**	0.91±0.23	0.63±0.16	0.5±0.1
	**Cu**	0.68±0.12	0.56±0.10	<0.60***
	**Zn**	2.83±0.48	1.27±0.22	1.0±0.2
	**Mn**	1.15±0.18	0.70±0.11	0.5±0.1
	**Fe**	**12.8±2.0**	**49.9±8.0**	**25.3±4.0**

(*) Result below lower Detection Limit (DL): for Cl = 10.0 mg/dm3
(**) Result above upper limit of the working range: for Mg = 400 mg/dm3
(***) Result below lower Detection Limit (DL): for Cu = 0.6 mg/dm3
(No. 1) Commercial soil (for sowing, quilting and herbs) containing a starting mild dose of fertilizer mixed with vermiculite (3:1)
(No. 2) Commercial de-acidified moss mixed with commercial soil containing a base dose of fertilizer and vermiculite (2:1:1)
